# In vitro investigation of osteogenic differentiation and bone regeneration potential of magnesium oxide nanoparticles incorporated into bacterial cellulose/silk fibroin scaffolds

**DOI:** 10.1038/s41598-026-50227-5

**Published:** 2026-04-26

**Authors:** Behrooz Niknafs, Parinaz Ahangar, Mohammadali Meskaraf-asadabadi, Elham Ghanbari

**Affiliations:** 1 Immunology Research Center, Tabriz University of Medical Sciences, Tabriz, Iran; 2 Australian Institute for Bioengineering and Nanotechnology, The University of Queensland, St Lucia, QLD 4072 Australia; 3 Department of Tissue Engineering, School of Medicine, Kermanshah University of Medical Sciences, Kermanshah, Iran; 4 Fertility and Infertility Research Center, Health Technology Institute, Kermanshah University of Medical Sciences, Kermanshah, Iran

**Keywords:** Bone regeneration, Magnesium oxide, Osteogenic differentiation, Bacterial cellulose, Silk fibroin, Nanoparticles, Biotechnology, Materials science, Stem cells

## Abstract

This study developed porous scaffolds composed of silk fibroin (SF), bacterial cellulose (BC), and magnesium oxide nanoparticles (MgONPs) via freeze-drying to investigate their potential for bone tissue engineering (BTE). The scaffolds were characterized using scanning electron microscopy (SEM) and Fourier transform infrared spectroscopy (FTIR), and assessed for porosity, compressive strength, swelling ratio, and degradation rate. Biological evaluations included cell attachment, proliferation, and osteogenic differentiation of human adipose-derived stem cells (hASCs). Results indicated that the incorporation of MgONPs influenced scaffold properties, leading to a decrease in pore size and swelling capacity (*p* = 0.001). MTT assay confirmed high cell viability across all scaffolds, with BC/SF/MgONPs demonstrating enhanced biocompatibility after 72 h (*p* = 0.016 vs. SF). Furthermore, BC/SF and BC/SF/MgONPs scaffolds exhibited minimal hemolysis, suggesting improved hemocompatibility. Alkaline phosphatase (ALP) activity and alizarin red S staining analyses revealed significantly increased osteogenic potential for BC/SF/MgONPs scaffolds compared to SF scaffolds (*p* = 0.027 and *p* = 0.002, respectively vs. SF). Consistent with these findings, BC/SF/MgONPs scaffolds led to a significant increase in the expression of early and late osteogenic markers, namely *RUNX2* (*p* = 0.001), *ALP* (*p* = 0.002), and *BGLAP* (*p* = 0.016). These findings demonstrate that BC/SF/MgONPs scaffolds may represent an effective system for promoting the osteogenic differentiation of hASCs and hold promise for BTE applications.

## Introduction

Bone tissue has a remarkable regenerative capacity; however, in cases of severe and extensive bone damage, natural healing is insufficient, even with pharmacological and surgical interventions. Currently, bone grafting is considered the primary treatment for severe fractures and non-healing bone tissue, although its use is limited by several challenges, such as sourcing suitable graft tissue and the potential for immunogenicity^1^. To address this predicament, bone tissue engineering (BTE) has emerged as a promising approach for treating bone defects. It involves culturing live cells with growth factors and other biomolecules on prefabricated scaffolds to promote bone repair. These cell constructs are subsequently transplanted in vivo to replace bone tissue at defect sites, possessing structural and physiological properties similar to those of native tissue^2,3^.

The proper selection of biocompatible and biodegradable polymers for tissue regeneration is vital for scaffold fabrication. Bacterial cellulose (BC) and silk fibroin (SF) have been shown to significantly enhance the structural and functional properties of engineered tissues^4^. SF is a natural polymer with a typical crystalline β-sheet-rich structure. Due to its high biocompatibility, low inflammatory response, biodegradability, cost-effectiveness, and particularly outstanding mechanical properties, SF stands out as an ideal candidate for various regenerative applications^5,6^ and has been successfully processed into various forms of scaffolds, including films, nanofibers, gels, and sponges, and has been widely applied in BTE^7,8^. Despite its numerous benefits, SF’s extreme fragility limits its use as a biomaterial, particularly in bone tissue. However, modifying SF with biopolymers and creating hybrid structures can improve its degradation rate and performance^9^.

BC also exhibits good biocompatibility, with higher mechanical strength and superior cellular compatibility^10^. Its three-dimensional (3D) networks enable absorption of large quantities of water or biological fluids, maintaining its hydrophilic character. It also possesses good tensile strength young’s modulus, as well as toughness comparable to that of spongy bone^4,11^. These properties make BC a suitable candidate for bone regeneration applications and a suitable polymer to combine with other materials^12^. However, one major drawback of using BC for TE applications is its pore diameter, typically around 10 μm, which significantly impedes cell penetration and migration during cell culture. This limitation can hinder the growth of new bone tissue and reduce the effectiveness of the scaffold in BTE. The development of a hybrid BC/SF scaffold has resulted in increased compressive strength and enlarged pore diameters of up to approximately 40 μm^4^. This system provides an extracellular matrix similar to natural bone tissue, thereby enhancing cell adhesion, proliferation, and differentiation to a greater extent, which promotes better bone regeneration. Additionally, the porous structure of the scaffold enhances the transport of nutrients and oxygen to cells, thereby significantly promoting cell growth and development^5,13^. Although BC/SF scaffolds have been successfully applied in tissue regeneration, they do not optimally enhance osteogenic differentiation of stem cells, which is critical for bone repair^5^. To overcome this limitation, incorporating osteogenic compounds into these biomaterials represents a promising strategy.

Magnesium oxide nanoparticles (MgONPs) are widely used, cost-effective inorganic fillers recognized as safe by the US Food and Drug Administration (FDA)^14^. MgONPs enhance the mechanical properties of polymeric scaffolds and support osteoblast attachment and proliferation. They also boost ALP activity, exhibit antibacterial effects, promote angiogenesis, stimulate the secretion of bioactive factors, and facilitate extracellular matrix (ECM) formation through the controlled release of magnesium ions^15^. Additionally, the decomposition of MgO helps to moderate the acidic conditions created by polyester degradation and reduces the associated inflammatory response. MgO also plays a role in regulating cell cycle progression, cell proliferation, and differentiation^16–18^.

This study presents, for the first time, an integrated platform that combines SF, BC, and MgONPs to address challenges in BTE. This tri-component system facilitates a comprehensive evaluation of scaffold architecture, material chemistry, and the osteogenic differentiation of human adipose-derived stem cells (hASCs) within BC/SF/MgONP constructs. In this design, MgONPs enhance mineralization kinetics and signaling; BC provides a hydrated, robust network; and SF contributes bioactivity and processability, resulting in a porous, mechanically suitable, and osteoinductive platform. The integrated assessment encompasses physical properties, cellular responses, and osteogenic gene expression, establishing a direct correlation between scaffold design and osteogenesis. Although conducted in vitro, these findings establish a foundation for in vivo validation, with an emphasis on optimizing pore architecture for angiogenesis and dynamic mechanical loading.

We developed a BC/SF scaffold incorporating MgONPs that not only demonstrates osteogenic activity but also promotes initial hASC attachment to the scaffold. Subsequently, we investigate the combined biological and osteogenic effects of this BC/SF/MgONP composition on hASCs, focusing on adhesion, viability, and early osteogenic markers.

## Results

### Characterization of MgONPs

**Fig. 1:**
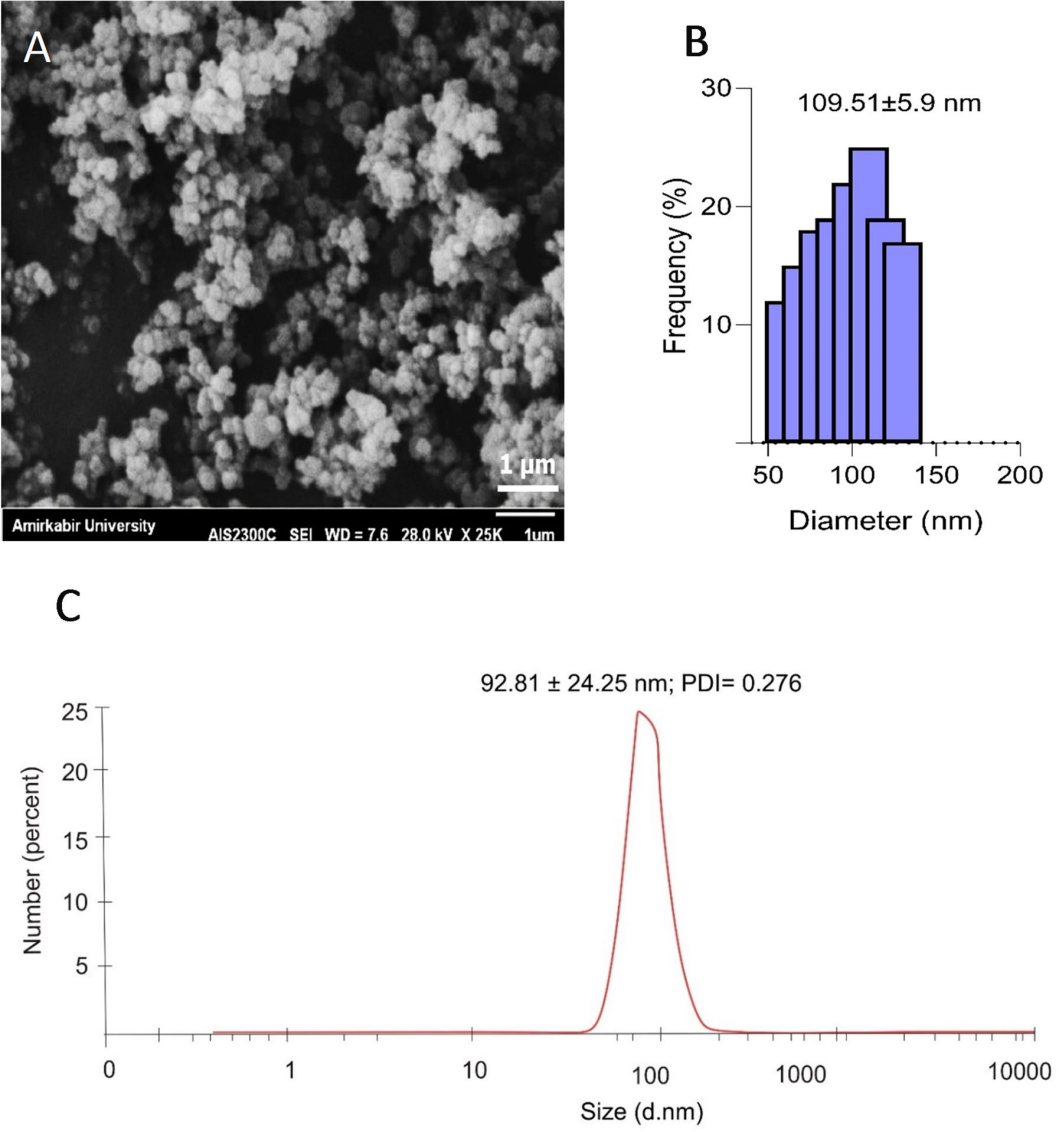

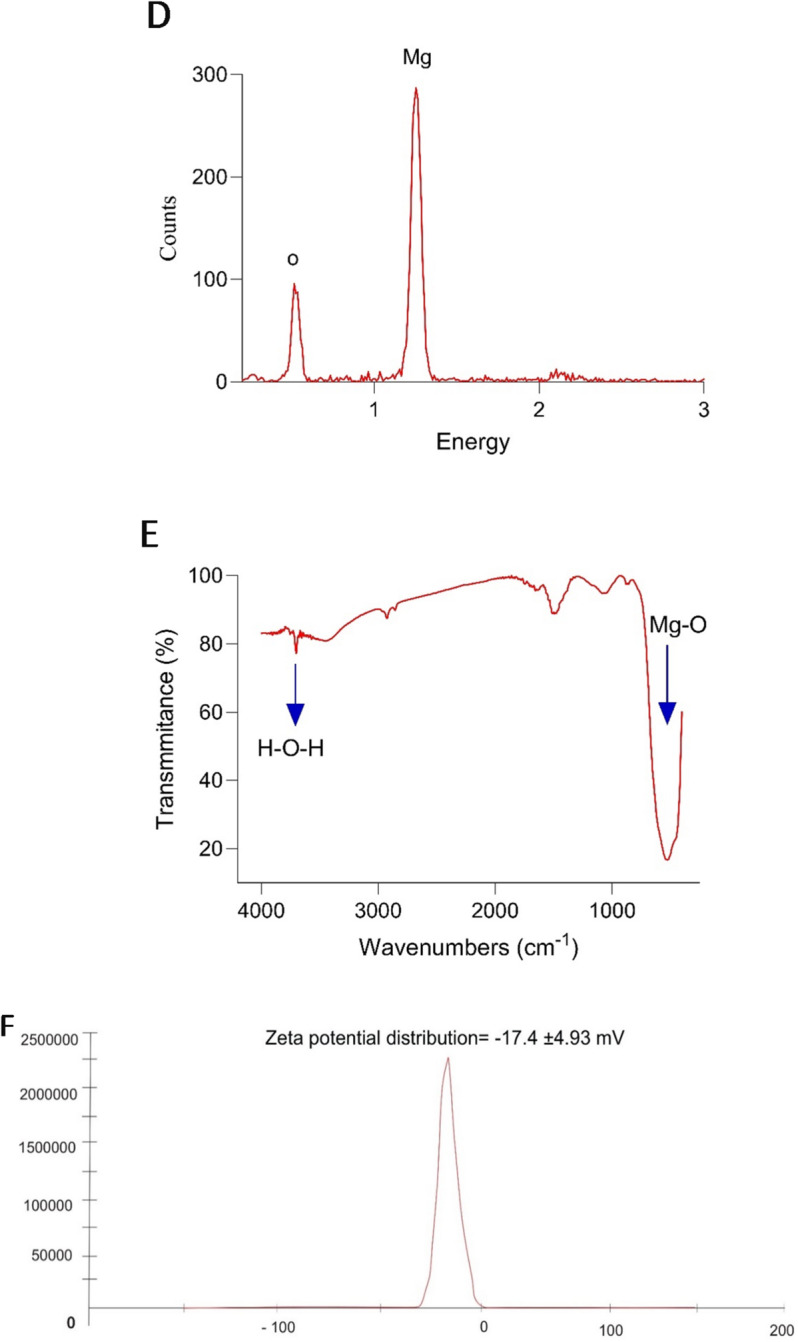
Characterization of magnesium oxide nanoparticles (MgONPs). (A) Scanning Electron Microscopy (SEM) image (scale bar=1 μm), (B) size distribution histogram of MgONPs obtained from SEM images; (C) particle size distribution of MgONPs obtained through Dynamic Light Scattering (DLS), (D) Elemental mapping of MgONPs was performed using EDX, (E) FTIR spectra of MgONPs, (F) Zeta potential measurements of MgONPs.

The morphology and corresponding histograms of the NP size distribution of MgONPs were analyzed using SEM. As illustrated in Fig. 1A and B, MgONPs exhibit a spherical morphology with an average diameter of 109.51 ± 5.9 nm. As demonstrated by the DLS analysis, the MgONPs exhibited uniform dispersion with a polydispersity index (PDI) of 0.276 and an average particle size of 92.81 ± 24.25 nm in DW (Fig. 1C). The EDX analysis of MgONPs revealed that magnesium (Mg) and oxygen (O) are the predominant elements (Fig. 1D).

Figure 1E presents the FTIR spectra of MgONPs. The peak at 522.71 cm^-1^ corresponds to the Mg-O stretching vibrations. The bands at 1634 cm^− 1^ and 3427.51 cm^-1^ are attributed to the bending and stretching vibrations of the hydroxyl group (-OH), respectively. Additionally, the absorption peak at 1490.97 cm^-1^ is ascribed to the stretching mode of the carbonyl group (C = O), which is associated with adsorbed carbonate (CO^-2^_3_) and carbon dioxide (CO_2_) species on the surface of MgONPs. Furthermore, the zeta potential results of MgONPs suspended in DW indicate a negative potential value of -17.4 ± 4.93 mV, indicating the stability of the MgONPs (Fig. 1F).

### Scaffolds characterization

**Fig. 2:**
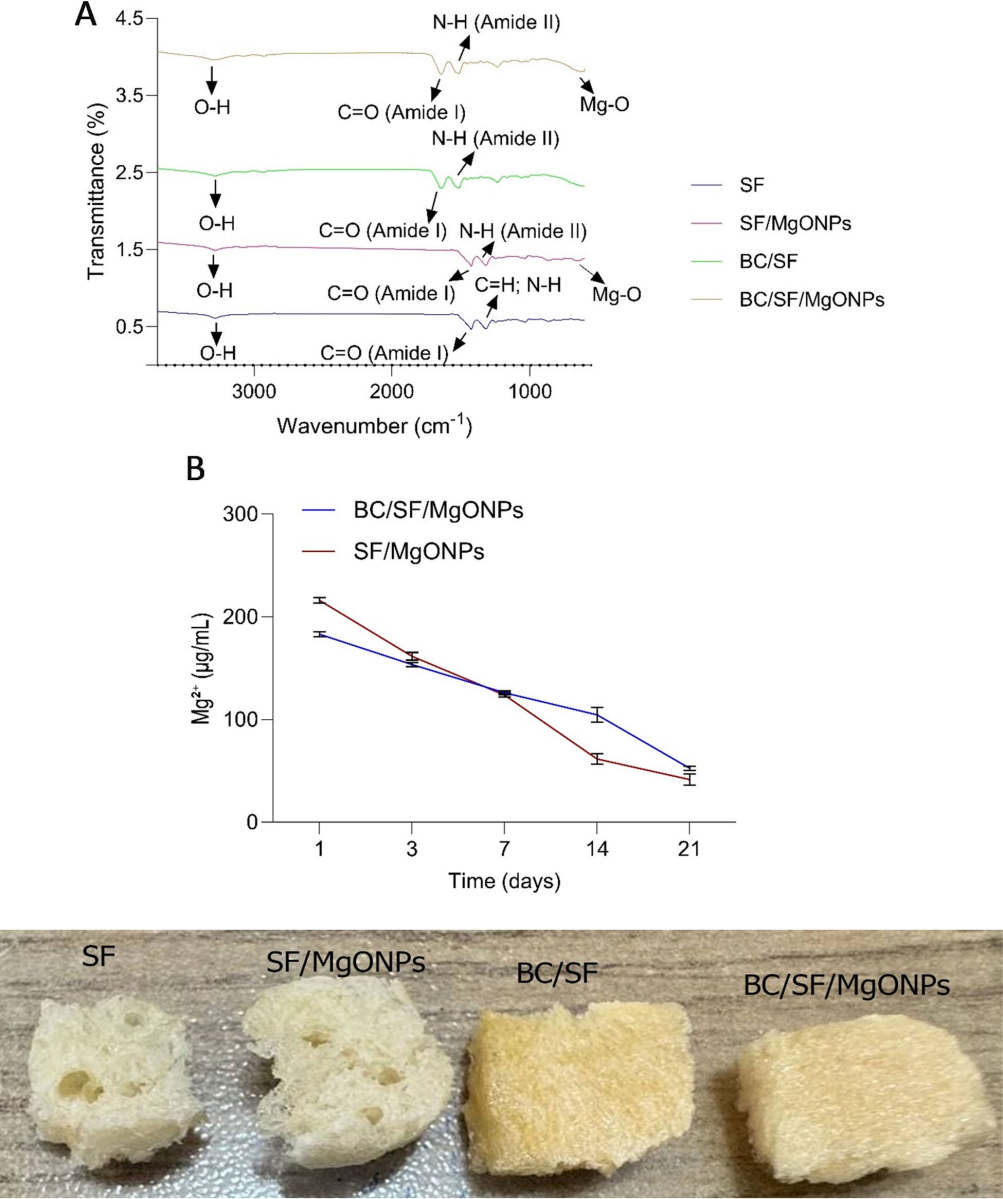

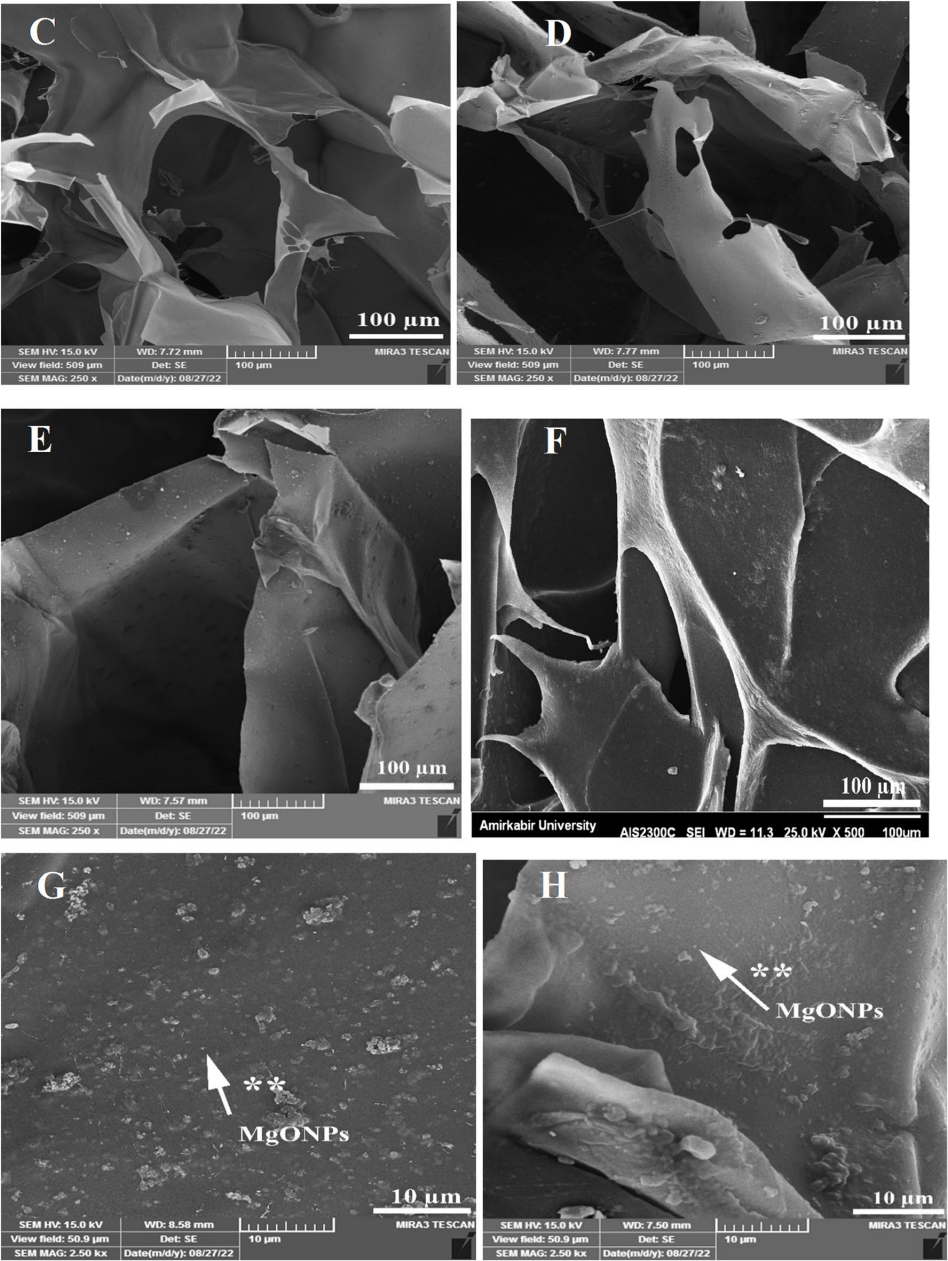
(A) FTIR results of SF, SF/MgONPs, BC/SF and BC/SF/MgONPs scaffolds and (B) Mg^2+^ release from BC/SF/MgONPs scaffold on the 1, 3-, 7-, 14- and 21-days experiments. Scanning Electron Microscopy (SEM) images of (C) SF scaffold (scale bar = 100 μm), (D) SF/MgONPs scaffolds (scale bar = 100 μm), (E) SF/BC scaffolds (scale bar = 100 μm), (F) SF/BC/MgONPs scaffolds (scale bar = 100 μm). The presence of MgONPs on the surface of the (G) SF/MgONPs (Scale bar=10 μm) and (H) BC/SF/MgONPs scaffolds (Scale bar=10 μm).

The FTIR spectra of SF, SF/MgONPs, BC/SF, and BC/SF/MgONPs scaffolds (Fig. 2A) exhibited characteristic amide bands. Peaks at 1630 cm^-1^ (amide I, C = O stretching) suggest the presence of random coil conformations, while the peak at 1556 cm^-1^, attributed to amide II (N-H bending) vibrations, indicates the presence of β-sheet structures. These findings suggest the coexistence of both β-sheet and random coil configurations within the scaffolds.

Furthermore, characteristic bands observed at approximately 500–620 cm^− 1^ in the SF/MgONPs and BC/SF/MgONPs samples are identified as Mg-O stretching vibrations, confirming the successful incorporation of MgONPs. For the BC-based scaffolds, prominent absorption peaks at 1013,1096, and 1121 cm^− 1^, associated with cellulose backbone vibrations, verify the integration of BC within the SF matrix. Finally, the broad absorption band from 3100 to 3500 cm^− 1^ is attributed to O-H stretching vibrations from the BC chains and SF amino acid residues. This peak signifies the presence of hydroxyl groups and the formation of a hydrogen-bonded network, which contributes to the hydrophilic nature of the scaffolds (Fig. 2A).

As depicted in Fig. 2B, the BC/SF/MgONPs composite demonstrated a sustained and prolonged release of Mg^2+^. The cumulative Mg^2+^ concentrations were measured at 183.1 ± 2.6 mg/L on day 1, gradually decreasing to 153.5 ± 2.2 mg/L by day 3, 126.1 ± 1.7 mg/L by day 7, 104.5 ± 7.3 mg/L by day 14, and 52.6 ± 2.1 mg/L by day 21.

In contrast, the SF/MgONPs scaffold exhibited a more pronounced initial burst release, starting at a significantly higher concentration of 216.2 ± 25 µg/L on day 1. However, this was followed by a rapid decline, reaching 161.71 ± 3.9 µg/L on day 3 and dropping sharply to 123.8 ± 2.2 µg/L and 61.7 ± 5.1 and 41.7 ± 5.5 µg/L by days 14 and 21, respectively.

In the SF scaffolds, SEM images revealed a porous structure with interconnected pores and an average pore diameter of 210 ± 10 μm. The thickness of the pore walls is uniform; however, the connectivity between the pores is poor, and the size and shape of the pores display nonuniformity (Fig. 2C). In contrast, the SF/MgONPs scaffolds demonstrated smaller pore sizes compared to the SF scaffolds, with a mean pore size of 175 ± 25 μm (Fig. 2D) and interconnected pores.

The BC/SF scaffolds showed a porous structure with uniform pore size, smooth surfaces, and well-connected pores, with diameters measuring 190 ± 15 μm (Fig. 2E). Notably, the BC/SF/MgONPs scaffolds exhibit a highly porous structure characterized by uniform pore size and good pore connectivity. However, the pore size and distribution vary across these scaffolds. Specifically, the BC/SF/MgONPs scaffolds present a porous structure with smaller pores measuring 115 ± 12 μm, while the SF, SF/MgONPs, and BC/SF scaffolds feature larger pores in comparison to the BC/SF/MgONPs scaffolds (Fig. 2F). MgONPs were observed on the surfaces of both SF/MgONPs (Fig. 2G) and BC/SF/MgONPs scaffolds (Fig. 2H).

**Fig. 3:**
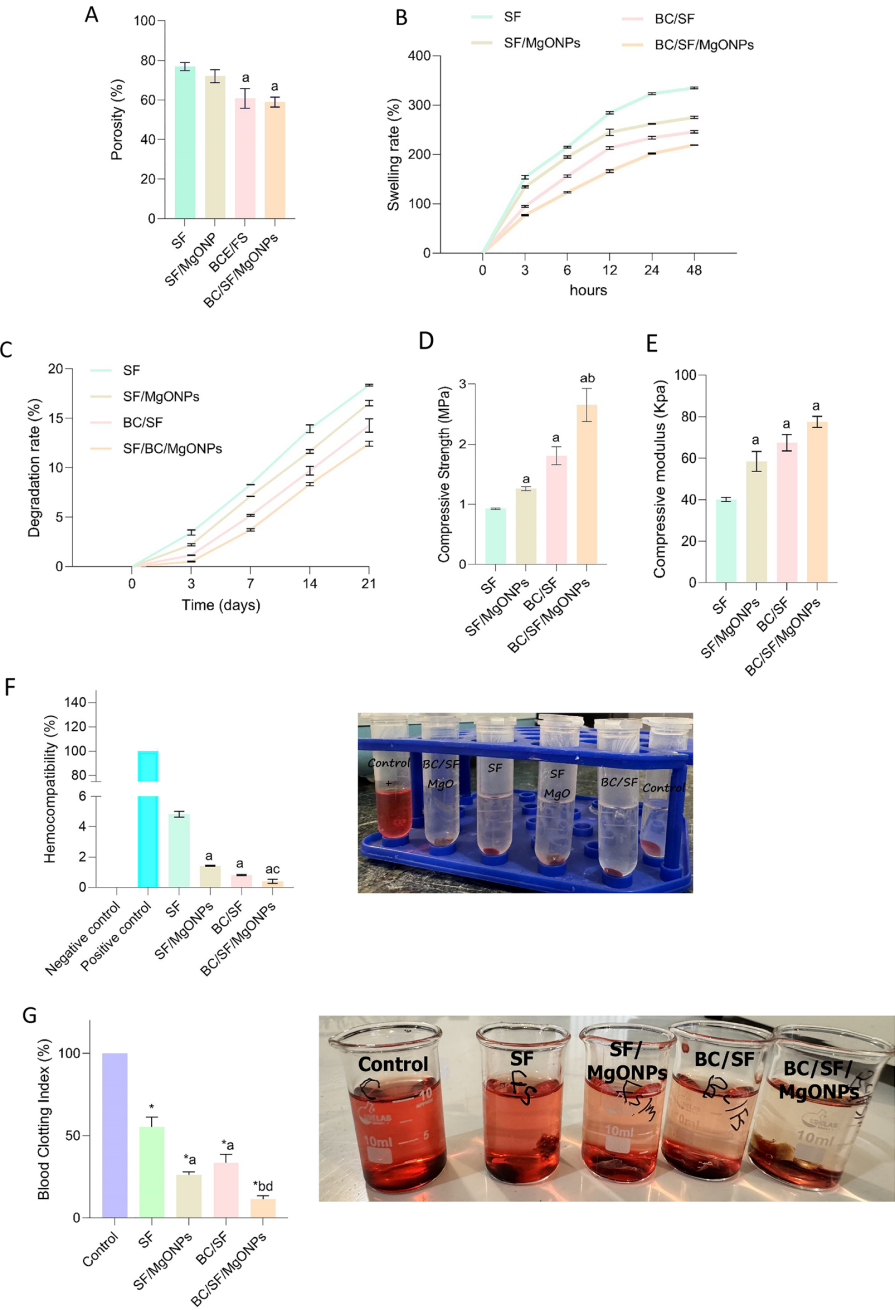
(A) Porosity, (B) swelling rate at 37 °C for 0, 3, 6. 12, 24 and 48 h in PBS, (C) degradation rate, (D) compressive strength, (E) compressive modulus of SF, SF/MgONPs, BC/SF, and BC/SF/MgONPs scaffolds (*n* = 3). (F) Hemocompatibility evaluation and (G) blood clotting for SF, SF/MgONPs, BC/SF and BC/SF/MgONPs scaffolds. *: Statistically significant difference compared with control group. a: Statistically significant difference compared with SF, *p* < 0.05; b: statistically significant difference compared with SF/MgONPs, *p* < 0.05. c: statistically significant difference compared with BC/SF/MgONPs, *p* < 0.05.

Furthermore, the influence of MgONPs on the porosity of SF and BC/SF scaffolds was investigated. The measured porosities for SF, SF/MgONPs, BC/SF, and BC/SF/MgONPs scaffolds were 76.9 ± 2.1%, 72.0 ± 3.2%, 60.8 ± 4.9%, and 58.9 ± 2.5%, respectively (Fig. 3A). Both BC/SF and BC/SF/MgONPs scaffolds showed significantly reduced porosity compared to the other groups (*P* = 0.015). Mean swelling rates for SF, SF/MgONPs, BC/SF, and BC/SF/MgONPs scaffolds were determined at 3, 6, 12, 24, and 48 h after incubation in PBS at 37 °C (Fig. 3B). After 48 h, the mean swelling rates were 334.7 ± 2.2%, 275.33 ± 2.3%, 245.67 ± 2.6%, and 219.3 ± 0.33%, respectively. The BC/SF/MgONPs scaffolds displayed a denser structure and smaller pore sizes compared to BC/SF scaffolds (*p* = 0.001; Fig. 3B).

Figure 3C illustrates the degradation rates of the scaffolds after 21 days of incubation in PBS at 37 °C. A significant reduction in degradation was observed for the SF/MgONPs, BC/SF, and BC/SF/MgONPs scaffolds compared with the SF scaffolds (*p* = 0.001). The recorded degradation values were approximately 18.3 ± 0.09%, 16.5 ± 0.3%, 14.3 ± 0.7%, and 12.4 ± 0.3% for the SF, SF/MgONPs, BC/SF, and BC/SF/MgONPs scaffolds, respectively. At all time points (days 3, 7, 14, and 21), the BC/SF/MgONPs scaffolds demonstrated the lowest degradation compared with the other groups.

Figure 3D demonstrates the mechanical strength of the scaffolds. The ultimate mechanical strength data for SF, SF/MgONPs, BC/SF, and BC/SF/MgONPs were recorded as 0.93 ± 0.01, 1.259 ± 0.03, 1.81 ± 0.15, and 2.65 ± 0.27 MPa, respectively. The Young’s modulus values were measured at 40.09 ± 0.97, 58.41 ± 4.85, 67.39 ± 3.94, and 77.51 ± 2.66 KPa, and 60.64 ± 8.67 MPa, respectively. These findings indicate that the BC/SF/MgONPs scaffolds exhibited significantly improved mechanical properties compared with the other groups (*p* = 0.001; Fig. 3E).

Figure 3F presents the percentage of hemolysis for each sample. The hemolysis percentages for the SF, SF/MgONPs, BC/SF, and BC/SF/MgONPs samples were 4.8 ± 0.18, 1.4 ± 0.33, 0.82 ± 0.04, and 0.41 ± 0.14, respectively. The percentage of hemolysis for the BC/SF and BC/SF/MgONPs groups was significantly lower than that of the SF scaffolds, suggesting enhanced compatibility with blood (*P* = 0.001). There was no significant difference in this parameter between the BC/SF and BC/SF/MgONP groups.

The BCI of the SF, SF/MgONPs, BC/SF, and BC/SF/MgONPs scaffolds were 55.2 ± 6.1%, 26.0 ± 2.0%, 33.5 ± 5.2%, and 11.5 ± 1.7%, respectively. As shown in Fig. 3G, the BC/SF/MgONPs scaffolds exhibited the lowest BCI among all scaffolds. In contrast, the SF and BC/SF scaffolds exhibited significantly higher BCI values compared with the SF/MgONPs and BC/SF/MgONPs scaffolds. Therefore, the incorporation of MgONPs into BC/SF and SF scaffolds significantly decreased the BCI percentage (*p* = 0.001).

### Biocompatibility of scaffolds

#### The effect of different scaffolds on hASCs viability

**Fig. 4:**
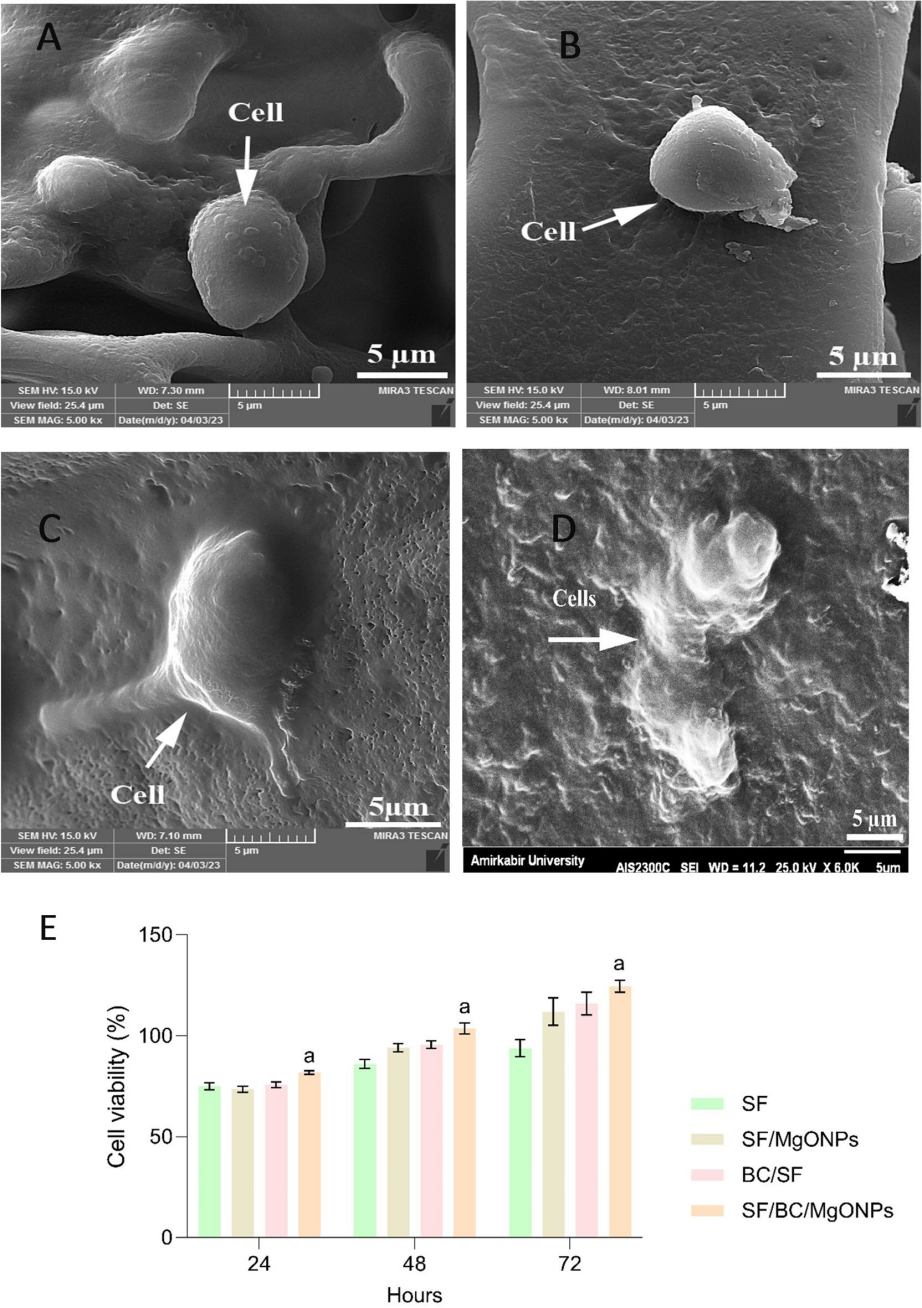
SEM images depicting the morphology of human Adipose-derived Stem Cells (hASCs) on SF, SF/MgONPs, BC/SF, and BC/SF/MgONPs scaffolds after 72 h of culture. (A) SF scaffolds (scale= 5 μm), (B) SF/MgONPs scaffolds, (C) BC/SF scaffolds (scale=5 μm), (D) BC/SF/MgONPs scaffolds (scale = 5 μm). (E) cell viability of hASCs on fabricated scaffolds 1, 2 and 3 days after seeding cell. a: Statistically significant difference compared with SF, *p* < 0.05.

Following 72 h of cellular seeding, cell adhesion was observed using SEM. hASCs on the SF scaffold exhibited a spherical morphology, indicating adhesion to the scaffold surface (Fig. 4A). Cells on SF/MgONPs scaffolds exhibited an elongated shape (Fig. 4B) and hASCs demonstrated attachment to the BC/SF scaffold surfaces, as shown in SEM images (Fig. 4C). The results indicate that the cells cultured on the BC/SF/MgONPs scaffolds displayed a spherical morphology (Fig. 4D).

The metabolic activity and viability of hASCs cultured on the various scaffolds were quantitatively assessed using the MTT assay at 24, 48, and 72 h (Fig. 4E). A consistent, time-dependent increase in cell viability was observed across all experimental groups, indicating that the fabricated scaffolds facilitated cell attachment and proliferation without inducing cytotoxic effects. At 24 h, cell viability for the SF, SF/MgONPs, and BC/SF groups was 74.98 ± 1.79%, 73.56 ± 1.50%, and 75.71 ± 1.41%, respectively. Notably, the SF/BC/MgONPs group exhibited a statistically significant increase in viability (81.77 ± 0.87%) compared to the SF group (*p* = 0.016). By 48 h, viability in the SF/BC/MgONPs group reached 103.67 ± 2.77%, which was significantly higher than that of the SF (86.10 ± 2.20%), SF/MgONPs (94.04 ± 2.03%), and BC/SF scaffolds (94.04 ± 2.03%) (Fig. 4E; *p* = 0.004).

After 72 h of co-culture, the relative cell viability values for the SF, SF/MgONPs, BC/SF, and BC/SF/MgONPs scaffolds were 93.85 ± 4.2, 111.9 ± 6.81, 115.9 ± 5.63, and 124.53 ± 3.03, respectively. The SF scaffold served as the control. Among the groups, cells cultured on the BC/SF/MgONPs scaffolds exhibited significantly higher viability and growth than those on SF scaffolds (*p* = 0.016; Fig. 4E). These findings indicate that the BC/SF/MgONPs scaffolds provide a more supportive microenvironment for hASC viability compared with SF or BC/SF scaffolds alone (Fig. 4E).

#### hASCs characterization

**Fig. 5:**
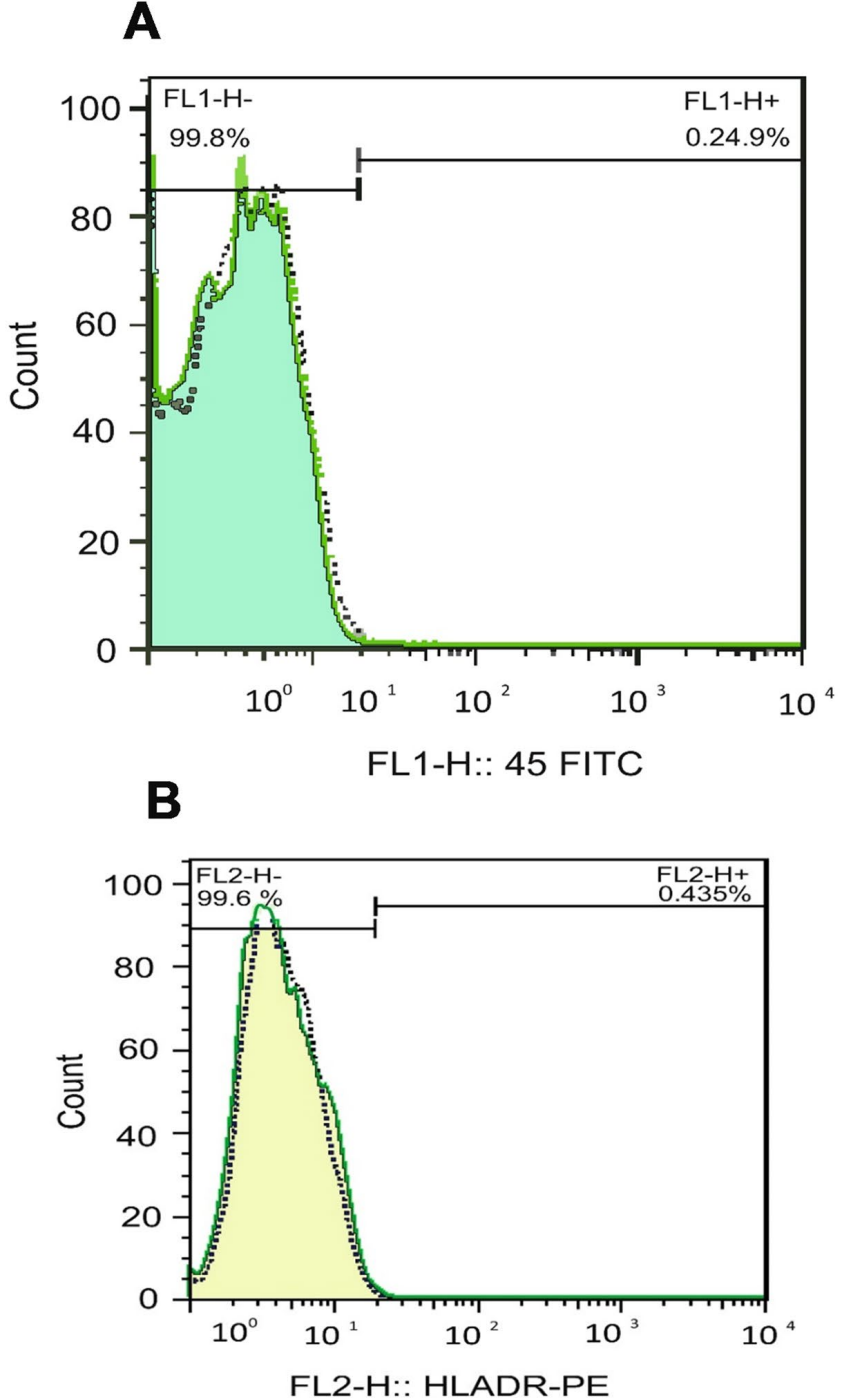

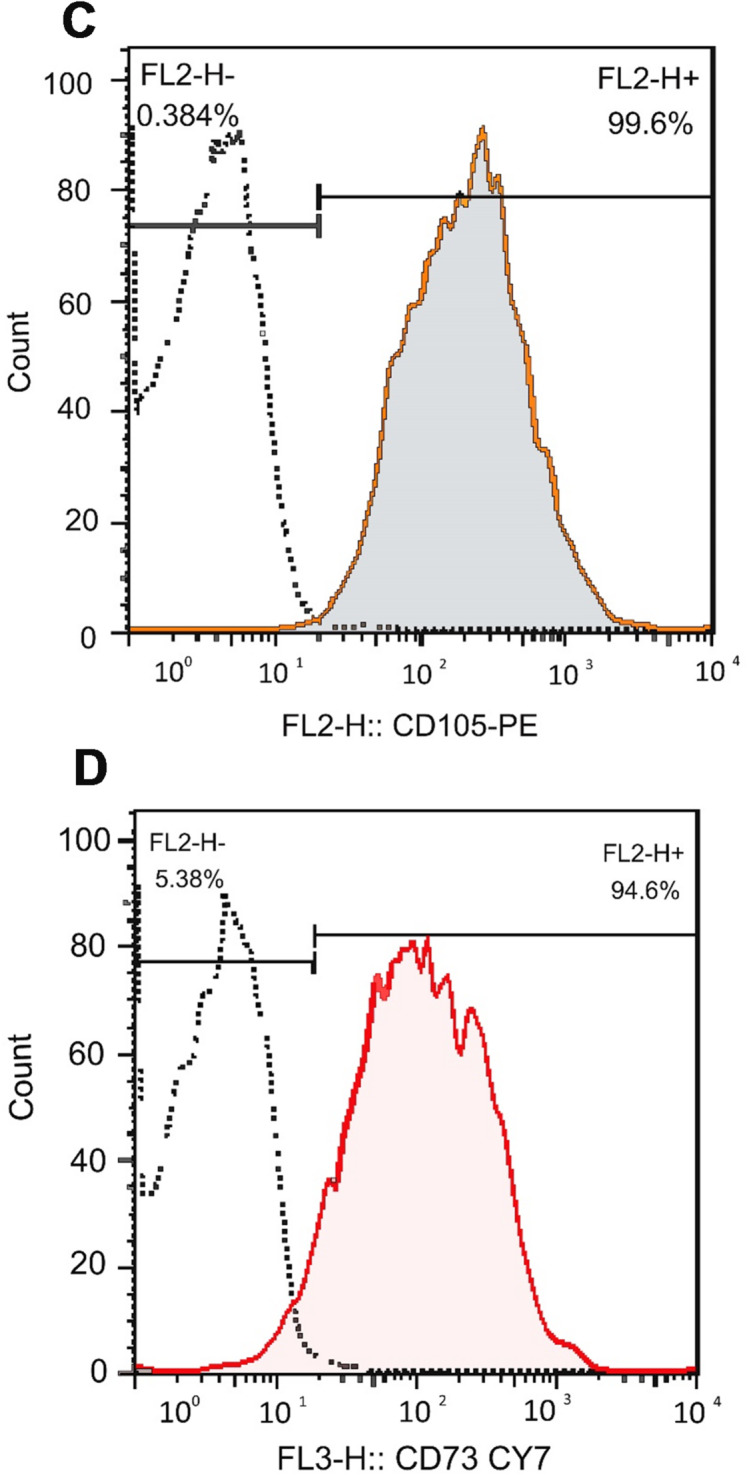

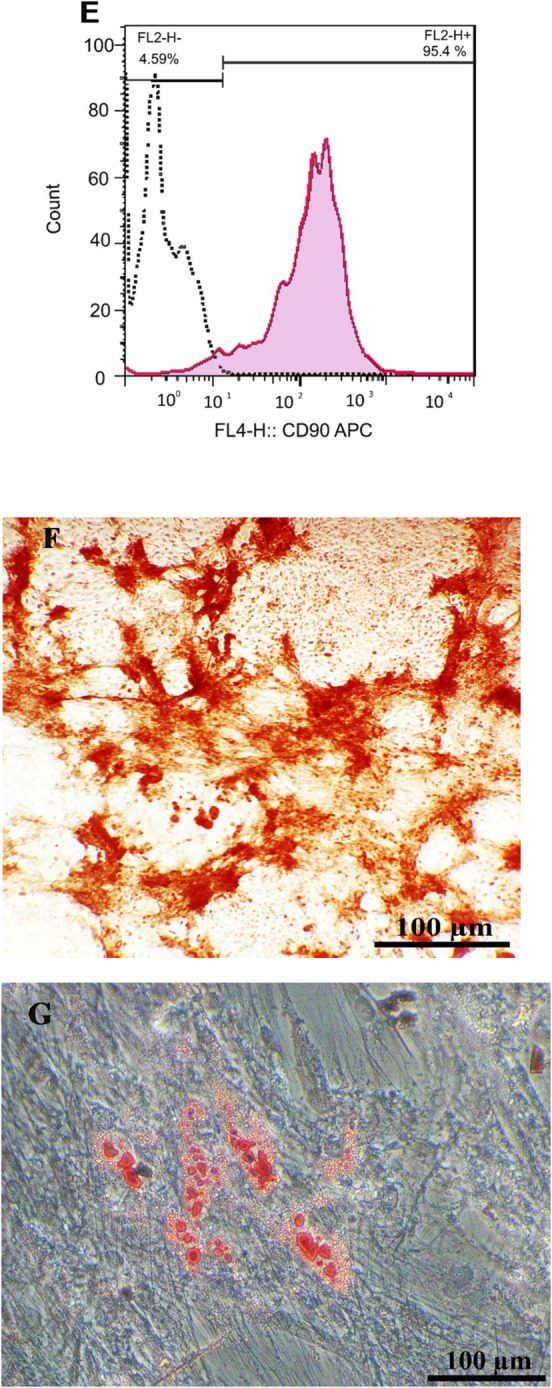
Flow cytometry results for markers of adipose tissue-derived mesenchymal stem cells (hASCs): (A) CD45 negative, (B) HLA-DR negative, (C) CD105 positive, (D) CD73 positive, (E) CD90 positive. Alizarin red staining assay on 21 days. (F) The calcified nodules showed a red color and (G) Oil Red O staining of hASCs following 21 days of treatment with adipogenic media revealed the accumulation of lipid droplets in the cytoplasm of differentiated adipocytes, which exhibited a red color with Oil Red O staining (scale bar = 100 μm).

Flow cytometry analysis of freshly isolated hASCs revealed expression of the surface markers CD73, CD90, and CD105, with levels of 94.6%, 95.4%, and 99.8%, respectively (Fig. 5A–C). Conversely, these cells exhibited a minimal expression of CD45 (99.8%) and HLA-DR (99.6%), confirming their non-hematopoietic phenotype (Fig. 5D and E).

Furthermore, the osteogenic and adipogenic differentiation potentials were confirmed by alizarin red staining (ARS) and oil red O staining, respectively. After 21 days of incubation, ARS staining revealed significant mineral deposition (Fig. 5F), while oil red O staining showed the formation of lipid vacuoles (Fig. 5G).

#### The effect of different scaffolds on hASCs attachment

**Fig. 6:**
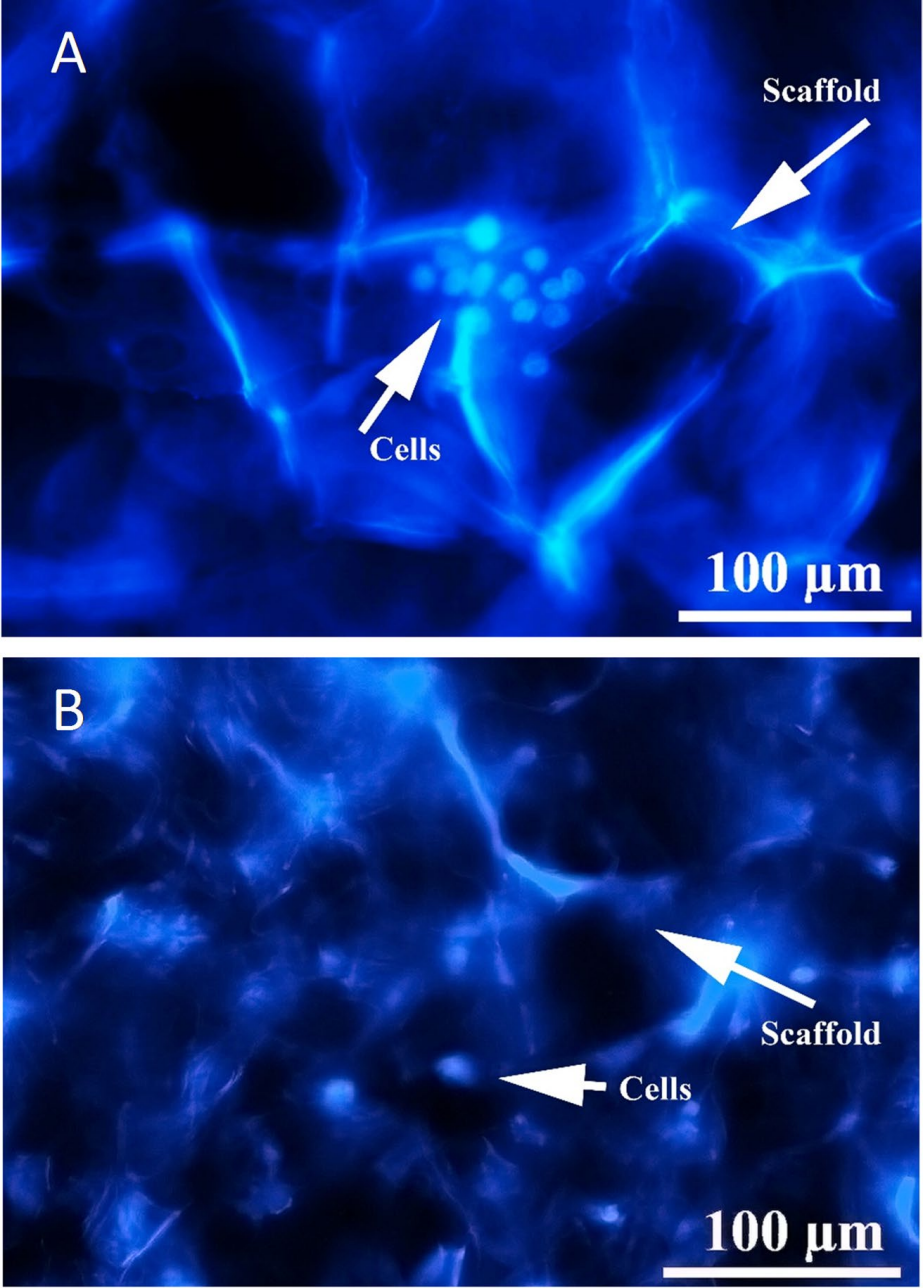

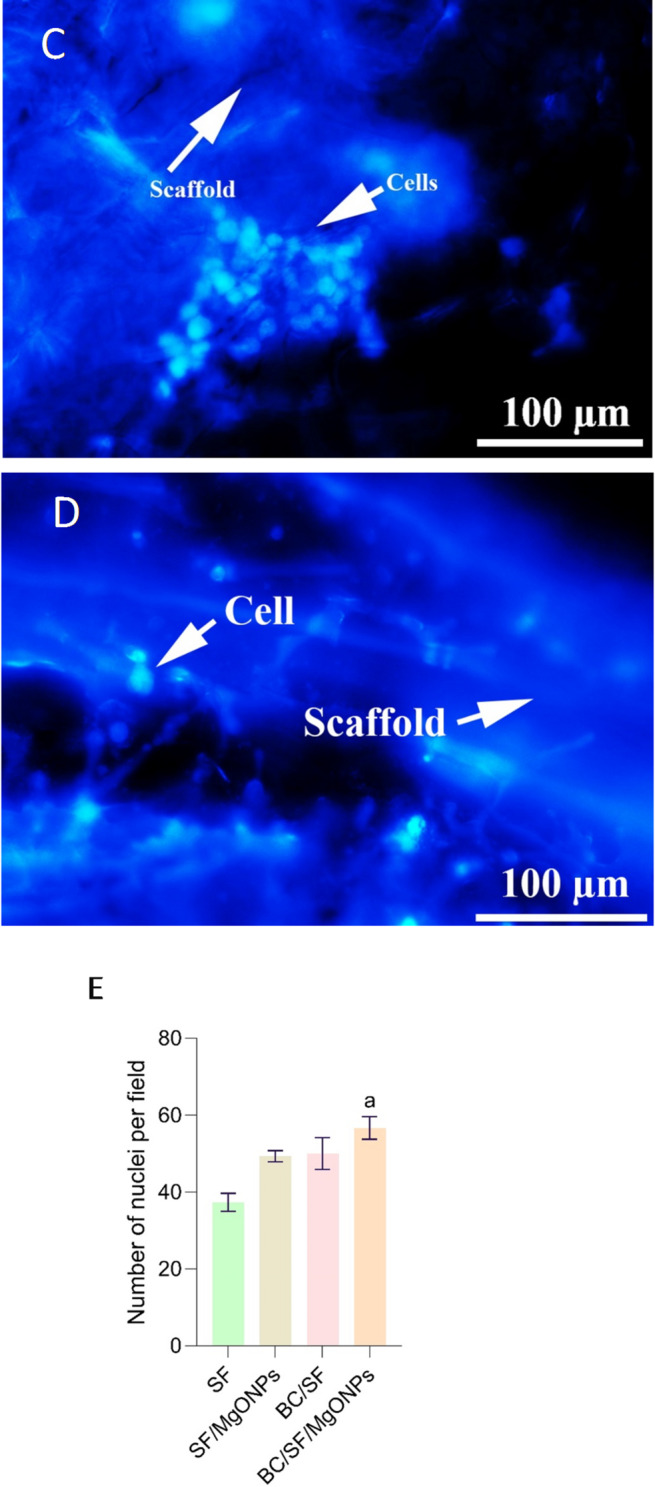
DAPI staining of the nucleus in hASCs cultured on the (A) SF, (B) SF/MgONPs, (C) BC/SF, and (D) BC/SF/MgONPs scaffolds after 7 days (scale bar = 100 μm). (E) Quantitative analysis of the number of nuclei in cells cultured on the scaffolds for 7 days. a: statistically significant difference compared with SF, *p* < 0.05; b: statistically significant difference compared with BC/SF, *p* < 0.05.

DAPI staining images, presented in Fig. 6 (A, B, C, and D), illustrate the distribution and density of hASCs nuclei on the various scaffolds after 7 days of culture. Quantitative analysis of these images (Fig. 6E) revealed that the number of cell nuclei (cell density) in hASCs cultured on SF/MgONPs, BC/SF, and BC/SF/MgONPs scaffolds was significantly higher compared to that observed on SF scaffolds. Specifically, the cell nucleus counts were 37.33 ± 2.3 nuclei per field (npf) for SF, 50.0 ± 4.16 npf for BC/SF, and 56.67 ± 2.96 npf for BC/SF/MgONPs scaffolds. These results suggest enhanced cell attachment on BC/SF and BC/SF/MgONPs scaffolds compared with the SF scaffold (*p* = 0.01), with BC/SF/MgONPs supporting the highest cell density.

#### The effect of different scaffolds on osteogenic differentiation of hASCs

**Fig. 7:**
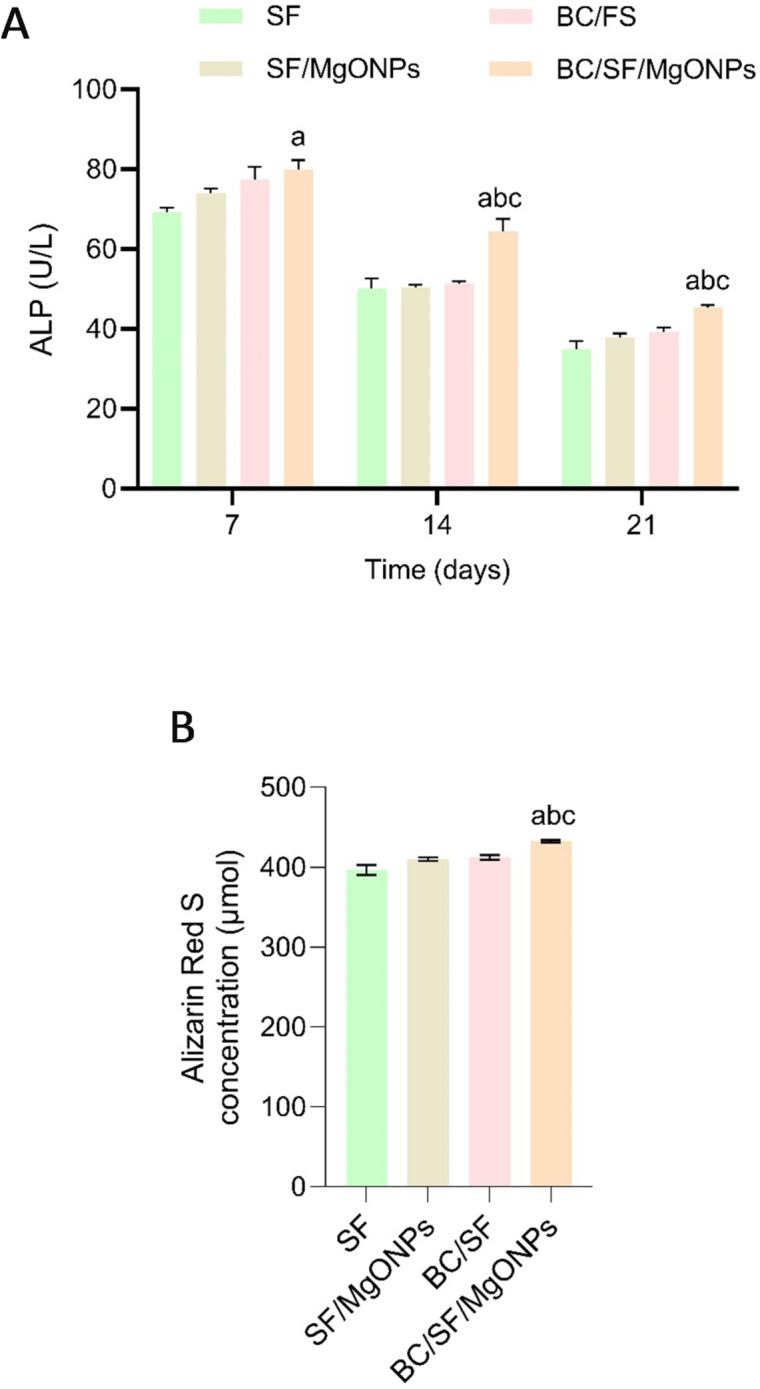
(A) ALP activity and (B) quantitative analysis of alizarin red s staining in hASCs cultured on SF, SF/MgONPs, BC/SF and BC/SF/MgONPs scaffolds for 21 days. a: Statistically significant difference compared with SF, *p* < 0.05; b: significant difference compared with SF/MgONPs, *p* < 0.05; c: statistically significant difference compared with BC/SF, *p* < 0.05.

Figure 7A presents the ALP activity for hASCs cultured on SF, SF/MgONPs, BC/SF, and BC/SF/MgONPs scaffolds. After 7 and 14 days of osteogenic differentiation, cells cultured on BC/SF/MgONPs scaffolds exhibited the highest ALP activity (*p* = 0.03). By day 21, the BC/SF/MgONPs group showed a significant increase in ALP activity compared with the other scaffolds (Fig. 7A; *p* = 0.001). Additionally, the highest levels of ALP activity were observed in the BC/SF/MgONPs group at day 7 (*p* = 0.027).

Quantitative ARS analysis demonstrated that calcium deposition was significantly higher in the BC/SF/MgONPs group than in the other scaffolds at day 21 (Fig. 7B; *p* = 0.002).

**Fig. 8:**
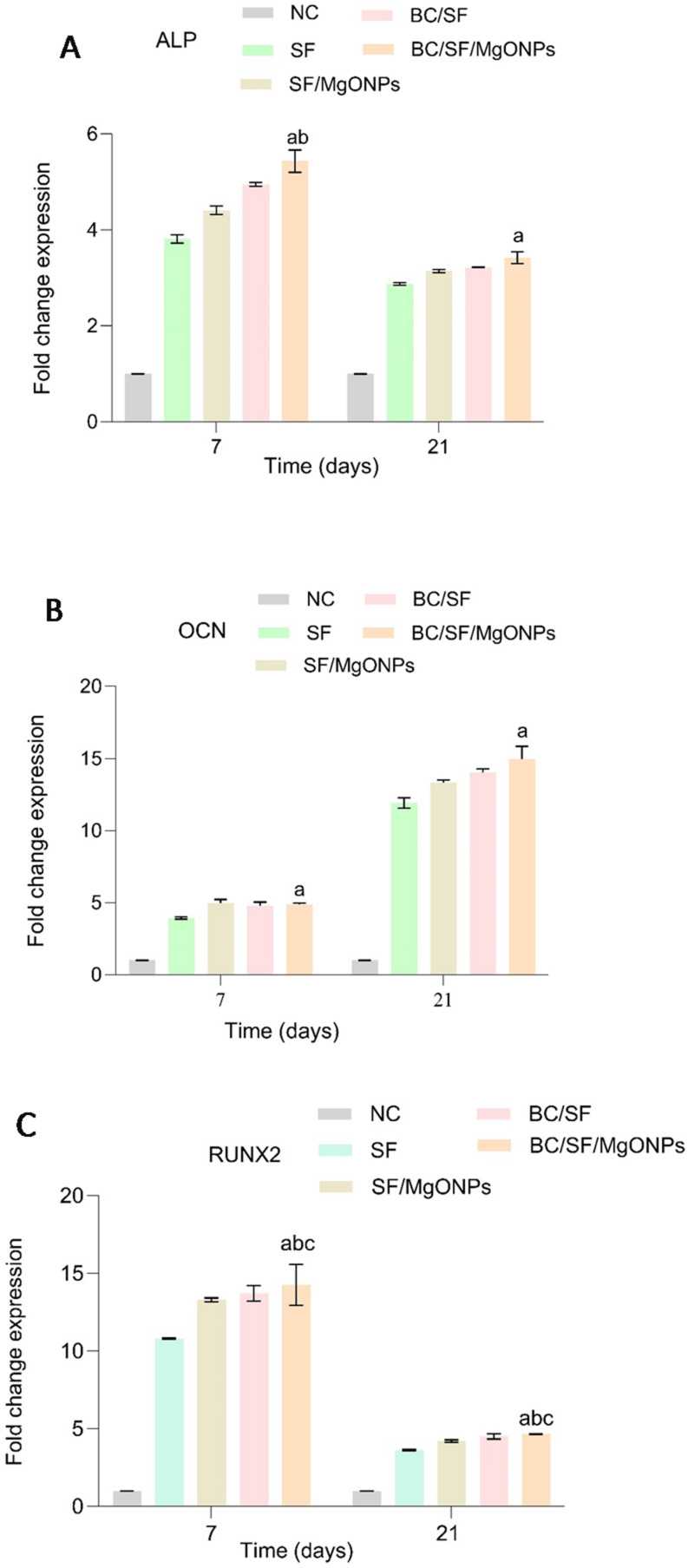
In vitro analysis of osteogenic gene expression in human adipose-derived stem cells (hASCs) cultured on different scaffolds. (A) *ALP* gene expression, (B) *BGLAP* gene expression, and (C) *RUNX2* gene expression in cells cultured on SF, SF/MgONPs, BC/SF, and BC/SF/MgONPs scaffolds. NC = negative control (The hASCs were cultured in α-MEM medium); a: Statistically significant difference compared with SF, *p* < 0.05, b: significant difference compared with SF/MgONPs, *p* < 0.05; c: statistically significant difference compared with BC/SF, *p* < 0.05.

Gene expression analysis revealed that *ALP* expression in cells cultured on BC/SF/MgONPs scaffolds was significantly increased at day 7, reaching its maximum, compared with the SF group (*p* = 0.001). While *ALP* expression remained significantly higher in the BC/SF/MgONPs group compared to SF and SF/MgONPs at day 21 (*p* = 0.002), its level at day 21 showed a decrease when compared to its peak at day 7 (Fig. 8A).

Bone gamma-carboxyglutamate protein (*BGLAP*), a key late marker of osteogenic differentiation produced by osteoblasts, showed significantly upregulated expression in the BC/SF/MgONPs group on days 7 and 21 (*p* = 0.024 and 0.016, respectively; Fig. 8B).

*RUNX2*, critical for early osteoblast development and matrix mineralization, was significantly upregulated in hASCs cultured on BC/SF/MgONPs scaffolds at day 7 compared with SF (*p* = 0.035; Fig. 8C). By day 21, *RUNX2* expression remained significantly higher in the BC/SF/MgONPs group relative to SF (*p* = 0.001; Fig. 8C).

## Discussion

In the present study, MgONPs with a diameter below 100 nm were successfully syntheiszed and incorporated into composite scaffolds, including SF, SF/MgONPs, BC/SF, and BC/SF/MgONPs. A variety of microscopic and spectroscopic techniques were employed to investigate the physicochemical and morphological characteristics of the NPs and scaffolds.

SEM analysis revealed that MgONPs have a spherical morphology with an approximate particle size of ~ 100 nm. The EDX results further confirmed the presence of Mg and O elements within the structure of the MgONPs. Additionally, the FTIR spectrum showed the characteristic band of MgONPs located in the range of 400 to 600 cm^–1^, which can be attributed to the Mg-O band. These features, consistent with similar MgONPs synthesis methods, are crucial for enhancing nanoparticle dispersion and interactions within polymeric matrices^15,19^.

FTIR spectroscopy was performed to characterize the interfacial interactions between BC, SF, and MgONPs. The broad absorption band observed at 3100–3500 cm^–1^, attributed to O-H stretching, indicates the presence of hydroxyl groups from the BC and SF amino acid residues. The persistence of this peak in the BC/SF/MgONPs composite suggests the maintenance of hydrophilicity and the formation of a hydrogen-bonded network, potentially facilitating nutrient transport and cell signaling, which are key factors in BTE.

Furthermore, the presence of amide I (C = O) and amide II (N-H) vibrations confirms that SF retains its structural integrity and stable conformation within the scaffolds. The emergence of the Mg-O stretching band at 500–620 cm^–1^ in the SF/MgONPs and BC/SF/MgONPs samples confirms the incorporation of the nanoparticles. These Mg-O groups may act as bioactive sites for ion release into the microenvironment. Biological data suggest that these ions, in coordination with the porous BC/SF scaffolds, contribute to the observed increases in ALP activity and calcium phosphate deposition (Alizarin Red S staining), supporting the osteogenic differentiation of hASCs on the BC/SF/MgONPs scaffolds.

In recent decades, researchers have increasingly focused on the development of suitable scaffolds for BTE, given their critical role in supporting regeneration. The design of these scaffolds is influenced by two primary factors: pore structure and mechanical strength. The mechanical strength must align with the compressive strength and modulus of the implantation area to adequately support cell adhesion and proliferation. Furthermore, the pore structure should be designed to be 3D and interconnected to promote the growth and migration of bone cells. A major challenge in this field lies in balancing pore size and mechanical characteristics, as excessively large and irregular pores may compromise the mechanical strength and overall effectiveness of the scaffold. In this study, BC/SF and BC/SF/MgONPs exhibited higher mechanical strength and Young’s modulus than SF scaffolds, with the BC/SF/MgONPs scaffold showing the highest values. These measurements are comparable to those of cancellous bone (compressive strength of 2–12 MPa and a compressive modulus of 0.02–5 GPa) (León et al.,[44]). The improved performance is attributed to the presence of BC, which generated smaller and more uniform pore structures and contributed to enhanced mechanical stability, confirming the successful incorporation of MgONPs into the BC/SF scaffolds.

Previous studies have reported that lyophilized SF sponge scaffolds exhibit brittleness and a sheet-like internal structure, which limits their utility in tissue engineering applications. To overcome these limitations, SF has been combined with other polymers, such as chitosan, to enhance mechanical strength^20^. Consistent with this approach, the incorporation of BC into SF in the present study substantially enhanced the mechanical strength, mitigating brittleness and improving overall scaffold performance. Moreover, the addition of MgONPs provided further mechanical reinforcement while simultaneously introducing bioactive functionality. The MgONPs significantly increased compressive strength and modulus while also providing Mg^2+^-mediated bioactive effects, such as modulation of cellular surface rigidity, stimulation of osteoblast activity, and promotion of angiogenesis and osteogenesis. In this composite system, SF provides biocompatibility and processability, BC offers a robust fibrillar and hydrophilic network, and MgONPs supply both mechanical and biochemical cues, together creating a multifunctional scaffold environment conducive to bone regeneration^21^.

Synergistic physical crosslinking also contributes to the enhanced mechanical integrity of BC/SF scaffolds loaded with MgONPs. SF crosslinking primarily occurs through the transition to stable β-sheet-rich structures, significantly induced by treatment with 90% methanol, which promotes extensive inter- and intramolecular hydrogen bonding and results in a robust, insoluble matrix^4^. Additionally, the interaction between BC and SF is driven by extensive hydrogen bonding between the abundant hydroxyl groups on BC and the amide and hydroxyl groups of SF, effectively tethering the SF matrix to the BC network and enhancing the overall cohesion of the composite^5^. Although MgONPs do not function as primary crosslinkers, they can influence β-sheet formation and establish additional physical interactions with both polymers, further stabilizing the composite structure. Collectively, these interactions form an integrated physical crosslinking framework that enhances mechanical performance and structural coherence.

Tissue engineering scaffolds are designed to mimic the extracellular matrix to support cellular function^22^. Ideal porosity, pore size and interconnectivity are fundamental for facilitating cellular infiltration, motility, and the circulation of oxygen and nutrients^23^. Although optimal pore size for osteogenesis remains context-dependent ranging from approximately 100–400 μm, some studies suggest that larger macroscopic pores can enhance osteogenic differentiation in certain collagen-based systems^4,16,24–26^. In the present study, freeze-dried BC/SF/MgONPs scaffolds exhibited interconnected pores with an average size of 115 ± 12 μm. While the incorporation of BC resulted in lower overall porosity compared to SF-based scaffolds, this denser architecture produced a more uniform and well-connected pore network.

Beyond porosity, the mechanical microenvironment plays a paramount role. As presented in Fig. 3A, our fabricated BC/SF and BC/SF/MgONPs scaffolds exhibited significantly lower initial overall porosities compared to the SF and SF/MgONPs groups. This reduction is primarily attributed to the incorporation of BC into the scaffold structure, which is known for its dense fibrillar network and high mechanical strength, leading to a more compact architecture^4^. The reduced overall porosity, smaller pore size, and higher stiffness observed in BC-containing scaffolds are therefore expected to provide favorable mechanical cues that promote osteoblastic commitment. In this context, the BC-enhanced scaffold architecture works synergistically with biochemical signals from MgONPs to support osteogenic differentiation and mineralized tissue formation.

The swelling behavior and in vitro degradation profiles of the scaffolds are presented in Fig. 3B and C, respectively. As illustrated in Fig. 3B, all scaffolds exhibited a rapid initial water uptake during the first 12 h, reaching a near-equilibrium state by 48 h. The SF scaffold demonstrated the highest swelling capacity at 335%, attributed to the abundant hydrophilic functional groups (hydroxyl, carboxyl, and amide) that facilitate hydrogen bonding with the aqueous medium^4,7^. Conversely, a systematic reduction in the swelling rate was observed with the incorporation of BC and MgONPs, with the BC/SF/MgONPs group exhibiting the lowest expansion at 219%. This decline is ascribed to the formation of a more compact and interconnected network. The interfacial interactions—specifically, hydrogen bonding between BC and SF, and electrostatic attractions between MgONPs and the polymer chains—enhance the effective cross-linking density. This structural refinement reduces the available free volume and restricts the mobility of the polymer chains, thereby limiting their capacity for water retention^27^.

The degradation analysis offers valuable insights into the structural stability of the developed scaffolds. During the 21-day incubation period, the weight loss exhibited a clear trend: SF > SF/MgONPs > BC/SF > BC/SF/MgONPs. The SF scaffold demonstrated the most significant degradation, while the BC/SF/MgONPs displayed a more controlled weight loss profile. This relative increase in biostability can be attributed to several factors. First, the incorporation of BC, recognized for its high crystallinity, likely provides a structural framework that enhances resistance to hydrolytic cleavage compared to SF. Second, the MgONPs may function as reinforcing fillers, potentially inducing physical tortuosity that slows the diffusion of the degradation medium into the scaffold. Additionally, the presence of MgONPs could help stabilize the local pH, thereby reducing the risk of accelerated hydrolysis often associated with localized acidic shifts^28^. The correlation between a denser microstructure, as indicated by the swelling data, and the presence of reinforcing phases appears to promote a more sustained degradation profile. Such controlled degradation is essential for biomaterials designed to provide temporary mechanical support during the gradual process of bone tissue regeneration.

Blood compatibility is a key requirement for implantable scaffolds. In this study, BC-containing scaffolds demonstrated enhanced blood clot formation, with complete coagulation observed on BC/SF/MgONPs scaffolds, likely due to the strong blood absorption capacity and platelet activation associated with BC^29^. Quantitative BCI analysis further revealed that SF scaffolds exhibited the highest BCI values, indicating slower clotting, whereas MgONP-containing scaffolds showed reduced BCI values. These results suggest that while BC promotes rapid clot formation, MgONPs modulate clotting behavior and reduce hemolysis, leading to improved hemocompatibility^30–32^. Consistent with this, BC/SF/MgONPs scaffolds exhibited the lowest hemolysis rates, well below the accepted threshold of 5%, confirming their blood compatibility^33^.

Cell viability and osteogenic differentiation were significantly enhanced on BC/SF/MgONPs scaffolds. MTT assays demonstrated higher cell survival compared to SF and BC/SF scaffolds, consistent with previous studies reporting the beneficial effects of MgONPs on biocompatibility^34,35^. hASCs also maintained their spherical shape when cultured in BC/SF, whereas those in BC/SF/MgONPs exhibited an elongated morphology. This cellular morphology suggests that SF and SF/MgONPs scaffolds exert minimal influence on the shape of hASCs, highlighting their biocompatibility and the preservation of cellular structural integrity. In contrast, BC/SF and BC/SF/MgONPs scaffolds induced elongated cell morphology, likely due to the hydrophilic properties of BC in these scaffolds^15^. Importantly, the positive effects of MgONPs are concentration-dependent; the selected nanoparticle loading in this study provided biological benefits without inducing cytotoxicity, indicating that Mg²⁺ release from the scaffold remained within a safe and effective range.

Over a 21-day incubation, the release kinetics of Mg^2+^ ions demonstrated a biphasic and sustained profile, characterized by an initial controlled burst phase followed by a prolonged, steady-state release. This sustained release is primarily attributed to the synergistic entrapment of MgONPs within the integrated BC/SF scaffolds, which modulates ion diffusion through the polymeric interstices^36^. This controlled delivery mechanism is critical for mitigating localized ion toxicity, a common limitation in metallic-based scaffolds, while ensuring persistent osteogenic stimulation, consistent with recent advancements in BC-based magnesium delivery systems^15,37^.

Comparative analysis underscores the pivotal role of BC in modulating the diffusion kinetics. In contrast to the SF/MgONPs, which exhibited rapid ion depletion due to high scaffold porosity and accelerated hydration of SF, the BC/SF/MgONPs composite demonstrated a near-linear release trajectory. This attenuation of release rates is attributed to the dense, interconnected fibrillar morphology of BC^4^, which enhances the tortuosity of the diffusion pathways, thereby impeding the outward flux of Mg^2+^ ions. Furthermore, the high density of hydroxyl functional groups on the BC backbone may facilitate transient electrostatic interactions with Mg^2+^ ions, effectively anchoring the nanoparticles and mitigating premature leaching. From a translational perspective, this sustained therapeutic window is instrumental for BTE, circumventing the cytotoxic thresholds associated with abrupt ion spikes and providing a stable microenvironment conducive to long-term osteoblast proliferation and biomineralization throughout the critical 21-day healing phase.

The incorporation of MgONPs significantly enhanced osteogenic differentiation of hASCs, as evidenced by increased ALP activity, mineralized nodule formation, and upregulation of osteogenic genes, including *RUNX2*, ALP, and *BGLAP*. *ALP* expression and activity peaked at day 7, followed by a gradual decline at later time points, while *BGLAP* expression increased significantly by day 21. This sequential expression pattern reflects the normal progression of osteogenic differentiation, where early ALP activity supports matrix maturation, followed by late-stage mineralization marked by *BGLAP* expression^38,39^. Enhanced *RUNX2* expression further suggests that Mg^2+^ released from MgONPs activates key osteogenic signaling pathways, including Wnt/β-catenin signaling^40^.

Consistent with these molecular findings, ARS staining at day 21 revealed significantly higher mineral deposition in BC/SF/MgONPs scaffolds, confirming effective late-stage osteogenesis. The concordance between gene expression, enzyme activity, and mineralization demonstrates that BC/SF/MgONPs scaffolds not only initiate osteogenic differentiation but also support complete maturation toward functional bone matrix formation. Together, these results highlight the strong osteoinductive potential of the developed composite scaffold for BTE applications.

While this study utilized external osteogenic supplements to promote differentiation, the significantly higher ALP activity and mineralization observed in the BC/SF/MgONPs scaffolds, compared to the BC/SF scaffolds cultured in the same inductive environment, suggest a contributory role of Mg²⁺ ions in enhancing the osteogenic process. Previous studies indicate that the sustained release of Mg^2+^ may modulate key signaling pathways, such as Notch and Wnt/β-catenin, which are critical for osteogenic commitment^41^. While our current experimental design emphasized the synergistic effects of the scaffold and differentiation media, future investigations are recommended to assess the scaffolds in growth media alone to more accurately evaluate their independent osteo-inductive potential.

## Conclusions

This study successfully demonstrated the synthesis of BC/SF/MgONPs scaffolds through lyophilization, achieving a pore size of approximately 150 μm. Results from the swelling behavior analysis, in vitro degradation studies, and mechanical strength assessments revealed significant differences between the SF and BC/SF/MgONPs scaffolds. The BC/SF/MgONPs and BC/SF scaffolds exhibited synergistic effects in promoting cell adhesion and proliferation compared with SF scaffolds. Furthermore, the BC/SF/MgONPs facilitated both early and late-stage osteogenic differentiation of hASCs. These findings underscore the importance of pore size and scaffold composition in bone regeneration. Scaffolds with larger pore sizes, such as the BC/SF/MgONPs scaffolds, demonstrated a superior capacity to support cell adhesion, proliferation, and differentiation, which are crucial for bone formation. This study offers promising insights into the development of bone regeneration strategies using well-designed hydrogel scaffolds, paving the way for future research to elucidate the underlying mechanisms, refine scaffold properties, and implement them in an in vivo setting.

### Limitations and future directions

Despite these promising findings, this study has several limitations that should be acknowledged. A primary constraint is the absence of in vivo evaluations, which prevents a comprehensive assessment of the scaffolds’ long-term osteogenic capacity and systemic biological functionality. Furthermore, while osteogenesis was confirmed through gene expression analysis (RT-qPCR) and mineralized matrix deposition (Alizarin Red S), this study did not quantify the translation of these markers into functional bone-related proteins, such as osteopontin and osteonectin. While mRNA levels provide crucial insights into early cellular commitment, they do not always linearly correlate with protein synthesis due to post-translational modifications. Additionally, resource constraints limited the use of advanced analytical methods, such as single-cell RNA sequencing or flow cytometry, which could have provided a more granular understanding of hASC phenotype shifts. Future studies should focus on Western blotting or ELISA to validate protein expression and incorporate in vivo models to confirm the clinical translatability of the BC/SF/MgONP scaffolds.

## Materials and methods

### Materials

The BC was purchased from the Nano biopolymer Pars Company (Tehran, Iran). MgCl_2_ 0.6 H_2_O (Cat# 105833, Sigma-Aldrich, Germany) and absolute ethanol (purity > 99%, Cat# 32205) were obtained from Merck (Merck Millipore, Darmstadt, Germany). Glutaraldehyde 50% (Cat# G7651), 3-(4,5-dimethylthiazol-2-yl)-2,5-diphenyltetrazolium bromide (MTT) (Cat# M5655), dimethyl sulfoxide (DMSO, Cat# D2650) and alizarin red staining solution (40 mM, Cat# TMS-008) were purchased from Sigma (Sigma-Aldrich, Germany). Fetal bovine serum (FBS, Cat# 16-000-044), Dulbecco’s Modified Eagle Medium High Glucose (DMEM, Cat# 10565018), trypsin/EDTA 0.25% (Cat# 25200056), penicillin-streptomycin (10,000 U/mL, Cat# 15140148) were provided from Gibco (Gibco Invitrogen, Waltham, MA, USA).

### Synthesis of MgONPs

MgONPs were synthesized using 0.2 M MgCl₂·6 H₂O and NaOH solutions. The MgCl₂·6 H₂O solution was added to NaOH and stirred at 70 °C for 90 min. The resulting mixture was then centrifuged and washed to remove impurities. To expedite drying, the precipitate was placed at 40 °C until completely dry. Finally, the obtained magnesium hydroxide powder was calcined at 500 °C in a furnace, yielding a white MgONP powder^15^.

### Preparation of SF solution and synthesis of SF scaffolds

**Fig. 9:**
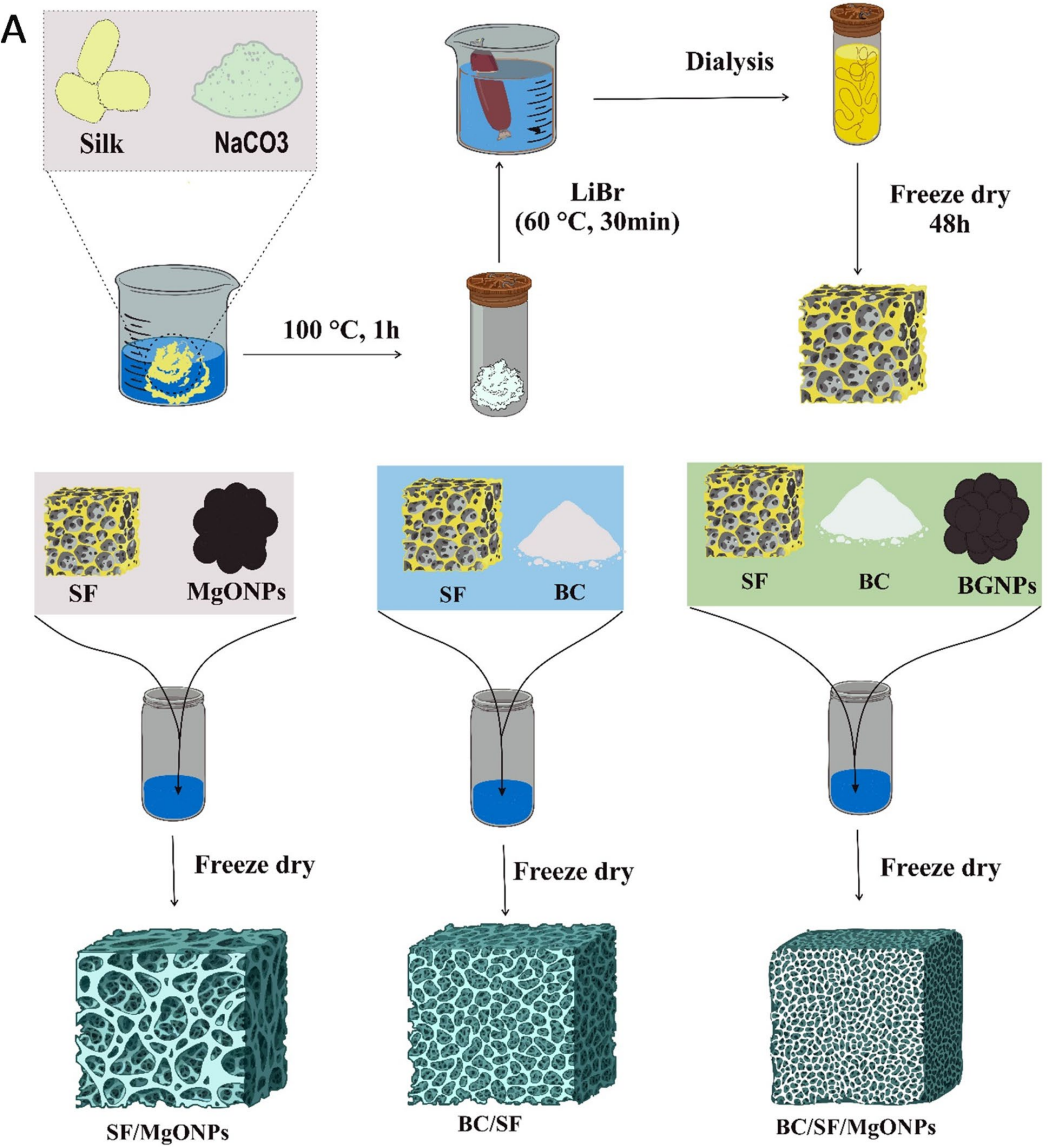

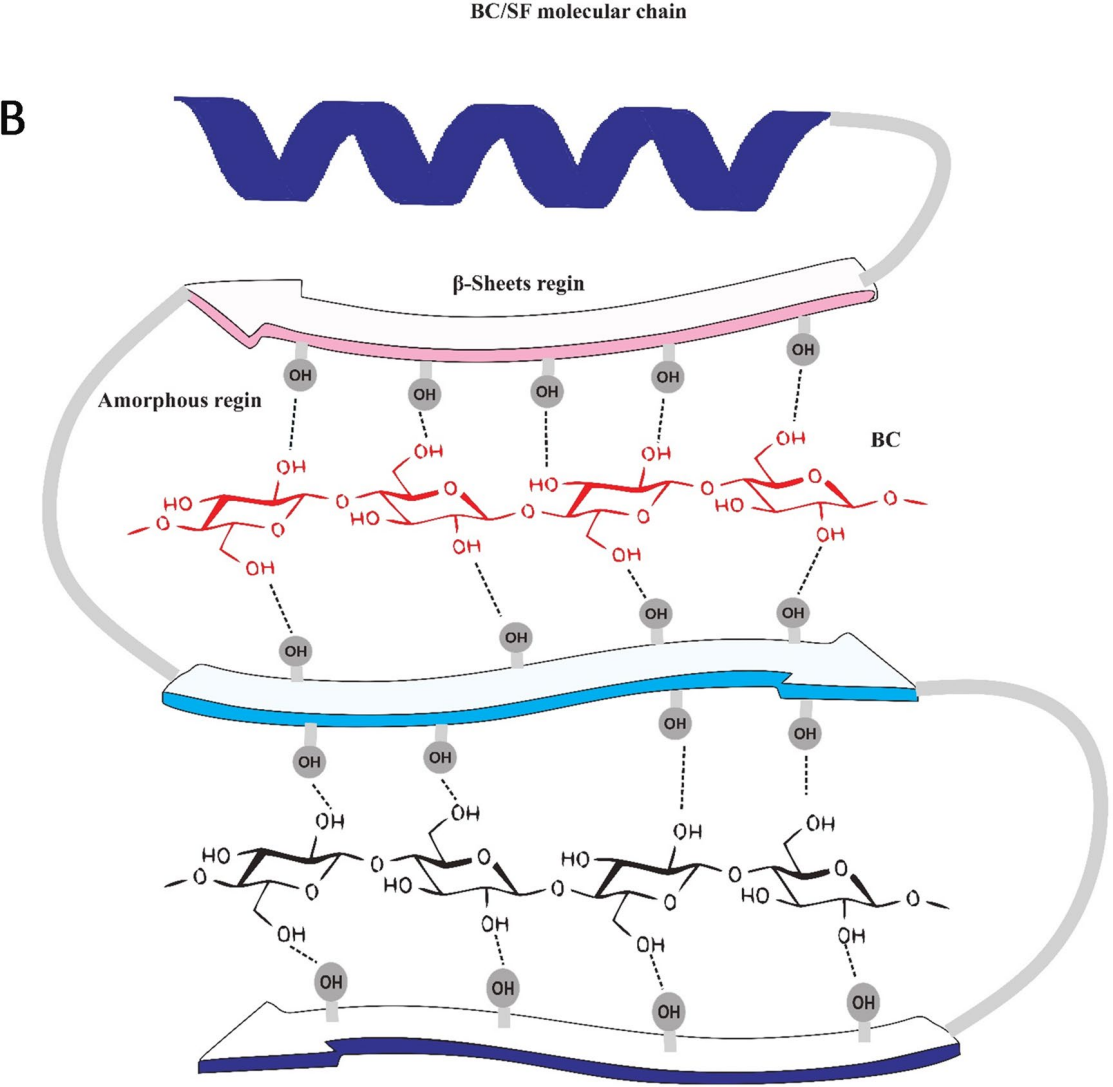
Schemes for preparation of SF, SF/MgONPs, BC/SF and BC/SF/MgONPs (A). Schematic illustration of the chemical structure of BC/SF scaffolds, (B) highlighting the hydrogen bond between BC and SF.

The SF aqueous solution was prepared as described in previous studies^42^. Briefly, the silk cocoons were cut into small pieces and boiled in 2% (w/v) Na_2_CO_3_ solution at 98 °C for 30 min to remove sericin. Then, the degummed fibers were rinsed three times with deionized water (DW) to remove any residual sodium carbonate (Na_2_CO_3_). Fibers were allowed to air-dry overnight at room temperature. Afterward, the SF was completely dissolved in a 9.3 M lithium bromide solution. Centrifugation at 9167 g (Centrifuge: SIGMA 1–15 PK, Germany) was performed for 20 min to eliminate impurities and any other undissolved particles from the formed solution. The SF was subsequently dialyzed against DW using a dialysis membrane (molecular weight cut-off of 10000) for 3 days. During dialysis, water exchange was performed at 1, 3, and 6 h, followed by every 12 h at 4 °C to prevent SF denaturation at higher temperatures. After dialysis, the SF solution was centrifuged at 9000 g (Centrifuge: SIGMA 1–15 PK, Germany) for 10 min, frozen at -20 °C for 24 h, followed by -80 °C for another 24 h, and finally lyophilized using a freeze-dryer (FD-5005-HS-BT, USA) for 48 h under vacuum (Fig. 9A and B).

### Preparation of SF, SF/MgONPs, BC/SF, and BC/SF/MgONPs scaffolds

To prepare SF scaffolds, 1.6 g of SF was dissolved in 40 mL of DW. To prepare SF/MgONPs scaffolds, 0.3 g of MgONPs was dissolved in a 100% SF solution. The BC/SF scaffolds were prepared by dissolving 0.2 g of BC powder (Nano Zist Polymer Pars, Iran) in 100 ml of 4% SF (w/v) solution. For the preparation of BC/SF/MgONPs scaffolds, 0.3 g of MgONPs powder was dispersed in 100 mL of the prepared BC/SF solution. Then, all scaffold solutions were frozen for 24 h at -20 °C and freeze-dried at 80 °C for 48 h. Finally, the obtained scaffolds were treated with 90% ethanol to prevent their dissolution in water (Fig. 9A, B).

### Mg ^2+^ release from the BC/SF/MgONPs composite scaffolds

To assess the release kinetics of magnesium ions (Mg^2+^), BC/SF/MgONPs were submerged in PBS. A consistent sample-to-solution volume ratio of 1:10 was maintained for all experiments. BC/SF/MgONPs composites were incubated at 37 °C for durations of 1, 3, 7, 14, and 21 days. The cumulative concentration of released Mg^2+^ ions was subsequently quantified using inductively coupled plasma optical emission spectrometry (ICP-OES/ICP-AES, ICP-6800, Chain).

### Characterization of MgONPs and scaffolds

#### Scanning electron microscopy (SEM)

For morphological investigation, powdered MgONPs and acellular scaffolds (BC/SF and BC/SF/MgONPs) were dried and mounted onto aluminum stubs with double-sided carbon tape, and sputter-coated with gold (Q150R S, Quorum Technologies, UK) for 90 s at 20 mA. Cell-seeded scaffolds were washed with PBS (pH 7.4) to remove non-adherent cells, fixed with 3.8% glutaraldehyde for 10 min at room temperature, and dehydrated in a graded ethanol series (25–100% v/v, 10 min each) before critical point drying and gold sputter-coating. Surface morphology was examined by field-emission scanning electron microscopy (FE-SEM; S-3000 H, Hitachi, Japan; MIRA3, Tescan, Czech Republic) at accelerating voltages of 15–25 kV, depending on the instrument. Particle size distribution of MgONPs was determined by analyzing ~ 100 particles from five randomly selected SEM images using ImageJ software (NIH, USA).

#### DLS, EDX and FTIR analysis of nanoparticles and scaffolds

A dynamic light scattering (DLS) particle analyzer was also used to determine the particle size distribution and surface charge of the nanoparticles (Malvern PANalytical Netherland). Energy-dispersive X-ray (EDX) analysis was used to determine the elemental composition of the MgONPs. Fourier-transform infrared spectroscopy (FTIR VERTEX70) was used to study the chemical bonds. The synthesized MgONPs and scaffolds were ground together with KBr powder for the FTIR spectra and then pressed into thin, transparent discs. The infrared spectra were recorded using 32 scans at a resolution of 4 cm^–1^ over the range of 400–4000 cm^–1^.

### Physicochemical characterization

#### Porosity measurement

The porosity of the fabricated scaffolds (SF, SF/MgONPs, BC/SF, and BC/SF/MgONPs) was determined using a liquid displacement method with absolute ethanol (99.9%). Initially, a known volume of ethanol (V1) was measured. A scaffold sample was then fully immersed in the ethanol for one hour to ensure complete liquid penetration. The combined volume of ethanol and the immersed scaffold (V2) was recorded. Subsequently, the scaffold was removed, and the remaining volume of ethanol (V3) was measured. The percentage porosity (%P) was calculated using the following formula:

$$\% {\text{P }}={\text{ }}\left( {{\text{V1 }} - {\text{ V3}}} \right){\text{ }}/{\text{ }}\left( {{\text{V2 }} - {\text{ V3}}} \right){\text{ }} \times {\text{ 1}}00\%$$

All measurements were performed in triplicate to ensure reproducibility.

#### Swelling study

The swelling rate for the fabricated scaffolds (SF, SF/MgONPs, BC/SF, and BC/SF/MgONPs) was measured at 0, 3, 6, 12, 24, and 48 h. The lyophilized samples were initially weighed and then soaked in phosphate-buffered saline (PBS) solution. The wet weight was measured at various time intervals by taking the samples from the solution and gently removing excess PBS with filter paper. Triplicate experiments were run under the same conditions for each scaffold. The swelling ratio percentage was calculated using the equation below, where Wd is the dry weight, and Ws is the weight in the swollen condition^5^.

$${\mathrm{Swelling~ratio~}}\left( {{\% }} \right)=\frac{{Ws - Wd}}{{Wd}} \times 100$$

#### Degradation study

The SF, SF/MgONPs, and BC/SF/MgONPs scaffolds were incubated in a 1% penicillin/streptomycin (pen/strep)-supplemented PBS (pH = 7.4) for 21 days under cell culture conditions of 95% relative humidity, 5% CO2 and 37 °C. At days 0, 3, 7, 14, and 21, the scaffolds were removed from the PBS solution, washed in deionized water (DW), dried in a vacuum drying oven, and then weighed. Four specimens were used for each scaffold type and time point^42^. The initial weight of each scaffold is marked as (Wc), and the weight of each scaffold at each time point is represented as (Wt). The degradation rate of the scaffold was assessed using the following formula:

$${\mathrm{Degradation~rate~}}\left( {{\% }} \right)=\frac{{\left( {{\mathrm{Wc~}} - {\mathrm{~Wt}}} \right)}}{{{\mathrm{Wc}}}} \times 100$$

#### Mechanical properties

The compressive strength of all scaffolds was measured using a universal testing machine (Zwick/Roell Z010, Germany) outfitted with a 20 N load cell. These test samples were in a cylindrical structure with dimensions of 1 cm × 1 cm. Scaffolds were placed between the jaws and compressed at a rate of 1 mm/min until failure occurred. The compressive modulus was calculated from the slope of the stress-strain curve using the appropriate equation:

$${\mathrm{E}}\,=\,\sigma /\varepsilon$$

σ: the compressive stress (Pa); ε: strain.

### Isolation of human adipose-derived mesenchymal stem cells (hASCs)

Fresh adipose tissue was obtained from five individual humans aged between 30 and 35 years (*n* = 5) undergoing liposuction. The adipose tissue was obtained with informed consent, and the procedure received approval from the Ethics Committee of Tabriz University of Medical Sciences (IR.TBZMED.REC.1400.376, dated July 26, 2021). All procedures were conducted in accordance with the most recent amendment of the declaration of Helsinki.

The isolated adipose tissue was first washed with sterile PBS containing a 2% solution of pen/strep antibiotics. The blood vessels and surrounding tissue were then carefully separated in a petri dish. The obtained adipose tissue was finely minced and subsequently mixed with 3% wt collagenase type 1 at a ratio of 1:20 (tissue to solution volume). The mixture of harvested fat and enzymes was incubated for 30 min at 37 °C in an incubator and then centrifuged for 5 min at 570 g (Centrifuge: SIGMA 1–15 PK, Germany). This centrifugation and washing process was repeated three times. The liquid supernatant was poured out, and the cellular pellet was reconstituted in Minimum Essential Medium Eagle-α-Modification (α-MEM: Cat. Number = BI-1010, BIOIDEA, Iran) supplemented with 1% pen/strep and 10% FBS. Consequently, the cell suspension was transferred to a culture flask and placed in an incubator with a controlled atmosphere of 5% CO2 and 95% humidity at 37 °C. Cell attachments were regularly observed, and changes in the medium were made every second day of the week. Cell morphologies were observed using phase-contrast microscopy. Once confluence reached 80–90%, the hASCs were treated gently with 0.25% trypsin/0.02% EDTA. Subsequently, the hASCs were expanded to passage three and then used in the subsequent experimental steps.

### Cell viability assay

The viability of hASCs cultured on SF, SF/MgONPs, BC/SF, and BC/SF/MgONPs scaffolds was assessed using the MTT assay. Briefly, 1 × 10^4^ cells were cultured either on tissue culture plates (control), or on SF, SF/MgONPs, BC/SF, and BC/SF/MgONPs scaffolds, each measuring 0.5 × 0.5 × 0.5 cm. On days 1, 2, and 3 of culture, the media were replaced with 500 µL of diluted MTT solution (400 µL culture medium and 100 µL of 5 mg/mL MTT in PBS).

The samples were then incubated in a CO_2_ incubator for 4 h. Following incubation, the solution was replaced with 500 µL of DMSO to dissolve the purple formazan crystals. Once the crystals had been completely dissolved, the solution was transferred to a 96-well plate for ELISA testing. Absorbance measurements were performed at a wavelength of 545 nm. Four samples from each group were analyzed.

$${\mathrm{Percentage~of~cell~viability~}}\left( {{\% }} \right)=\frac{{Absorbance{\mathrm{~}}of{\mathrm{~}}sample}}{{Absorbance{\mathrm{~}}of{\mathrm{~}}control}} \times 100$$

## Hemocompatibility evaluation

The biocompatibility of SF, SF/MgONPs, BC/SF, and BC/SF/MgONPs scaffolds was assessed using the hemolysis assay. Briefly, 2.50 mL of fresh anticoagulated human blood was mixed with heparin and diluted with 5 mL of normal saline (NS). The isolated red blood cells (RBCs) were centrifuged at 7,500 g (Centrifuge: SIGMA 1–15 PK, Germany). for 5 min, washed three times with NS, and diluted to the final volume of 20 ml. Cells were then treated with scaffolds (600 µg/mL) and incubated 37 °C for 3 h. Following incubation, samples were centrifuged, and 100 µL of the upper layer was transferred to a 96-well plate for determination. The absorbance of each well was measured at 577 nm using a microplate reader. The hemolysis percentage was calculated according to the following equation^43^:

$${\text{Hemolysis~\% }}=\frac{{{\mathrm{OD}}s - {\mathrm{OD}}nc}}{{2{\mathrm{OD}}pc - {\mathrm{~}}O{\mathrm{D}}nc}} \times 100$$

In this equation, ODs represents the sample’s absorbance value, whereas OD nc represents the absorbance value of the negative control (NS), and ODpc represents the absorbance value of the positive control (DW).

### Blood clotting index (BCI)

BCI tests were performed to investigate the coagulability of whole blood in vitro using SF, SF/MgONPs, BC/SF, and BC/SF/MgONPs scaffolds. A lower value indicates a good pro-coagulant effect. Samples were placed in a water bath maintained at 37 °C for an hour. Subsequently, 300 µL of anticoagulant-treated blood was added to each scaffold. After a 5-min incubation period, 25 µL of a 0.2 M CaCl_2_ solution was added. Twenty-five milliliters of DW were added to the samples 5 min later. Following a 5-min incubation at 37 °C, absorbance was measured at 542 nm. The control group consisted of 300 µL of blood and 25 ml of DW without any scaffold. The BCI was calculated using the following equation:

$${\mathrm{BCI}}\,=\,{\text{OD sample}}/{\text{OD control }} \times {\mathrm{1}}00\%$$

### Cell nuclear staining

4,6-Diamidino-2-phenylindole (DAPI) staining is a technique used for observing nuclei. Briefly, hASCs were cultured on SF, SF/MgONPs, BC/SF, and BC/SF/MgONPs scaffolds at a density of 10^4^ cells per sample. The scaffolds were maintained for 7 days under standard cell culture conditions in α-MEM supplemented with 10% fetal bovine serum (FBS) at 37 °C and 5% CO_2_. The scaffolds were then rinsed with PBS to eliminate nonadherent cells. The cells on the scaffolds were fixed with 4% formaldehyde for 15 min, permeabilized with 0.1% Triton X-100 in PBS for 1 min, and then incubated with a DAPI nuclear stain solution at a concentration of 1 µg/mL in PBS for 5 min. Finally, the fluorescent microscope (Olympus BX53) was used to capture images of the DAPI-stained samples, and after that, the nuclei count was carried out in six randomly chosen fields. The average nuclei count in these fields is the respective count of nuclei per field^5^.

### hASCs characterization

#### Flow cytometry analysis

Flow cytometry analysis was conducted to characterize the phenotypic profile of hASCs using cells at passage 3. Initially, hASCs were detached from culture flasks using 0.25% trypsin-EDTA, collected and washed twice with PBS by centrifugation at 200 g (Centrifuge: SIGMA 1–15 PK, Germany) for 5 min. The final pellet was resuspended in 1 mL of PBS containing 1% bovine serum albumin (BSA) to create a single-cell suspension. For immunophenotyping, 100 µL aliquots of the cell suspension were incubated for 30 min at 4 °C in the dark with the following specific fluorochrome-conjugated monoclonal antibodies (mAbs) at a concentration of 5 µL per tube:

- Anti-CD45-FITC (Cat: 304005, BioLegend).
- Anti-HLA-DR-FITC (Cat: 307604, BioLegend).
- Anti-CD90-APC (Cat: 328113, BioLegend).
- Anti-CD105-PerCP/Cyanine5.5 (Cat: 323215, BioLegend).
- Anti-CD73-PECY7 (Cat: 344009, BioLegend).

Following incubation, cells were washed with 500 µL of PBS and centrifuged at 200 g (Centrifuge: SIGMA 1–15 PK, Germany) for 5 min to remove unbound antibodies. The pellet was then resuspended in 250 µL of PBS, and data acquisition was performed using a BD FACS Calibur (BD biosciences, San Jose, CA). Subsequent data analysis was conducted using FlowJo software (FlowJo v10.10, China).

#### Adipogenic differentiation potential of hASCs

After 21 days of adipogenic differentiation, the accumulation of intracellular neutral lipids in differentiated adipocytes was assessed using the Oil Red O staining method. In summary, cells were fixed in 4% paraformaldehyde for 10 min and then rinsed three times with PBS. Specimens were subsequently incubated in a 0.25% w/v oil red O solution in 60% isopropanol for 10 min. Negative controls comprised hASCs cultured in a standard medium. Therefore, the Oil Red O staining technique allows the visualization of lipid droplets in differentiated adipocytes. This approach is a reliable indicator of the successful adipogenic differentiation of the hASCs, as the accumulation of neutral lipid droplets is a characteristic of adipocytes.

#### Osteogenic differentiation assay in hASCs in two-dimensional culture and hASCs seeded on scaffolds

To assess the osteogenic potential, all biological assays were performed using a 3D culture system. The SF, SF/MgONPs, BC/SF, and BC/SF/MgONPs scaffolds were fabricated (1 × 1 × 1 cm^3^) were sterilized using UV irradiation for 1 h. Given the substantial volume and high porosity of these 3D constructs, hASCs at passage 3 were seeded onto each scaffold. Following an initial attachment period, osteogenic differentiation was induced over a 21-day period using α-MEM supplemented with 10% FBS, 1% penicillin/streptomycin, 0.01 µM dexamethasone, 10 mM sodium β-glycerophosphate, and 50 µg/mL L-ascorbic acid-2-phosphate.

#### Alizarin red staining and quantitative analysis

Alizarin red S (ARS) staining was used to examine the osteogenic differentiation of hASCs. Briefly, hASCs in different scaffolds were cultured in the osteogenic differentiation medium for 21 days.

They were then rinsed with PBS (pH 7.4) and fixed for 20 min in a 4% paraformaldehyde solution.

After fixation, samples were stained with 0.2% (w/v) ARS (pH = 4.1) at room temperature for 15 min. The stained cells were visually examined and imaged using an inverted microscope (Eclipse-TE2000-S, Nikon, Japan).

For quantitative analysis, a 10% acetic acid solution was added to the cell-seeded scaffolds and stirred for 30 min. Subsequently, the solution was heated to 85 °C for 10 min, cooled and centrifuged at 9000 g (Centrifuge: Premium 20000 R, Iran) for 15 min.

The absorbance of the obtained red solution was measured using a spectrophotometer at a wavelength of 405 nm.

#### Determining ALP activity

Alkaline phosphatase (ALP) activity was evaluated to assess osteogenic differentiation of hASCs at 7, 14, and 21 days. Measurements were performed using a commercial ALP kit (Pars Azmoun, Iran) following the manufacturer’s instructions. The obtained ALP activity values were normalized to the total protein content of each sample.

#### Gene expression analysis

To assess the impact of SF, SF/MgONPs, BC/SF, and BC/SF/MgONPs on the osteogenic differentiation of hASCs cultured on these scaffolds, the expression levels of osteogenesis-related markers, including bone gamma-carboxyglutamate protein (*BGLAP*), *ALP*, and runt-related transcription factor 2 (*RUNX2*), were measured using quantitative real-time PCR (qRT-PCR) at both 7 and 21 days of culture. The scaffolds were washed with PBS, after which 1 mL of TRIzol reagent was added to the homogenized samples. This step disrupts the structural integrity of the scaffold and releases its cellular contents. Subsequently, 300 µL of cold chloroform (4 °C) was added to the homogenized sample, and the mixture was centrifuged at 15,000 g (Centrifuge: SIGMA 1–15 PK, Germany) for 15 min at 4 °C. The clear supernatant containing RNA was carefully decanted, and an equivalent volume of cold isopropanol was added. The sample was stored at -20 °C for 45 min to facilitate RNA precipitation. Following this, the sample was centrifuged for 15 min at 15,000 g at 4 °C. The liquid above the microtubes was discarded, and 1 mL of cold 75% ethanol was gently added to the sediment. The samples were centrifuged at 6,000 g (Centrifuge: SIGMA 1–15 PK, Germany) for 10 min at 4 °C to purify the precipitated RNA. The ethanol was then gradually removed, and the microtube was allowed to air-dry. Finally, nuclease-free water was added to the resultant pellet, and the sample was placed in a dry bath at 65 °C for 2 h under N_2_ atmosphere, followed by centrifugation at 12,000 g for 15 min. The RNA concentrations were measured using a Nanodrop spectrophotometer (Thermo Scientific, Germany, Deutschland). The iScript cDNA synthesis kit was used to synthesize cDNA from the isolated total RNA. The SYBR Green PCR Master Mix was used for quantitative PCR (qPCR). For data normalization, the glyceraldehyde-3-phosphate dehydrogenase (*GAPDH)* gene served as a reference.

Quantitative real-time PCR was conducted using cDNA, gene-specific forward and reverse primers, and Amplicon master mix (AMPLICON, Stenhuggervej, Denmark). The reactions were executed on a StepOne Real-time PCR system (Applied Biosystems, USA) with the following amplification protocol: an initial denaturation step of 15 min at 95 °C, followed by 40 cycles comprising denaturation at 95 °C for 15 s, and annealing and extension at 60 °C for 60 s.

Relative quantification of gene expressions for *RUNX2*, *ALP*, and *BGLAP* was calculated using the comparative Ct formula (2^–ΔΔCt^). All gene expression analyses were performed in quadruplicate, with hASCs cultured in standard growth medium (*n* = 5) serving as the negative control for osteogenic differentiation. The primer sequences used are listed in Table 1.

**Table 1:** List of human primer sequences for ALP, *BGLAP*, *Runx*2 and *GAPDH* gene.

Gene	Primer	Sequence (5′ → 3′)
*ALP*	Forward	GCTGGGAAATCTGTGGGC
	Reverse	CCATGATCACGTCAATGTCCCT
*Runx2*	Forward	TCATGGCGGGTAACGATGA
	Reverse	GGGAGGATTTGTGAAGACGG
*BGLAP*	Forward	CAGCCACCGAGACACCATGA
	Reverse	CTTGGACACAAAGGCTGCAC
*GAPDH*	Forward	AAGGTGAAGGTCGGAGTCAAC
	Reverse	GGGGTCATTGATGGCAACAA

*ALP*: Alkaline phosphatase; *Runx2*: Runt-related transcription factor 2; *BGLAP*: bone gamma-carboxyglutamate protein; *GAPDH*: Glyceraldehyde-3-phosphate dehydrogenase.

### Statistical analysis

All quantitative results are presented as mean ± SD, and the quantitative experiments were repeated at least in triplicate. Significant differences between groups were compared using one-way ANOVA using SPSS 26.0 statistical software. The statistical significance level (*p* < 0.05) was determined based on a one-way ANOVA, followed by Tukey’s post hoc test to identify specific group differences. Each condition was tested with five biological replicates (*n* = 5) to helps ensure the reliability and reproducibility of the results.

## Data Availability

The data presented in current study are available upon request from the corresponding author.
